# Implantable Passive Sensors for Biomedical Applications

**DOI:** 10.3390/s25010133

**Published:** 2024-12-28

**Authors:** Panagiotis Kassanos, Emmanouel Hourdakis

**Affiliations:** School of Electrical and Computer Engineering, National Technical University of Athens, 15772 Athens, Greece; p_kassanos@hotmail.com

**Keywords:** implantable sensors, passive sensors, inductive coupling, ultrasonic coupling, radiative coupling, galvanic coupling, capacitive diaphragm

## Abstract

In recent years, implantable sensors have been extensively researched since they allow localized sensing at an area of interest (e.g., within the vicinity of a surgical site or other implant). They allow unobtrusive and potentially continuous sensing, enabling greater specificity, early warning capabilities, and thus timely clinical intervention. Wireless remote interrogation of the implanted sensor is typically achieved using radio frequency (RF), inductive coupling or ultrasound through an external device. Two categories of implantable sensors are available, namely active and passive. Active sensors offer greater capabilities, such as on-node signal and data processing, multiplexing and multimodal sensing, while also allowing lower detection limits, the possibility to encode patient sensitive information and bidirectional communication. However, they require an energy source to operate. Battery implantation, and maintenance, remains a very important constraint in many implantable applications even though energy can be provided wirelessly through the external device, in some cases. On the other hand, passive sensors offer the possibility of detection without the need for a local energy source or active electronics. They also offer significant advantages in the areas of system complexity, cost and size. In this review, implantable passive sensor technologies will be discussed along with their communication and readout schemes. Materials, detection strategies and clinical applications of passive sensors will be described. Advantages over active sensor technologies will be highlighted, as well as critical aspects related to packaging and biocompatibility.

## 1. Introduction

Technological advancements over the last couple of decades in microelectronics, fabrication technologies, materials and telecommunications have led to the development of a wide range of biomedical devices [1,2]. These have led to an explosion in wearable devices that have managed to become commercial realities and are nowadays ubiquitous. Nevertheless, most of these devices are not considered suitable for diagnostic purposes and are thus primarily marketed for sport enthusiasts, athletes and people interested in monitoring a limited number of physiological parameters. Significant effort is still needed towards the development of wearables that can also provide data useful to physicians for diagnosis and prognosis, but that also provide a greater range of sensing capabilities. Such efforts have led to the demonstration of epidermal and lab-on-skin devices [3,4]. These advancements are motivating research in the field of implantable devices [2]. The prospect of implantable devices has fueled science fiction for decades yet, despite this, only a few commercial examples exist today. Perhaps the most successful and well-known are implantable devices interfacing with electrogenic tissue, such as the heart and the peripheral and central nervous systems. The implantable electrical pacemaker is the most popular implantable device, first demonstrated in 1958 [5]. Since then, the technology has evolved significantly, with several companies producing cardiac pacemakers around the world. This has led to devices for hearing restoration (cochlear implants), deep brain stimulation devices for tremor suppression related with Parkinson’s disease, and implantable spinal cord stimulators for pain management. Significant academic and industrial effort has been geared toward the development of neural interfaces for human-machine interfacing and for paraplegics to restore movement [6,7,8,9,10,11]. What these devices have in common is that they are meant to cure a condition and are primarily responsible for stimulating tissue, with some recording of action potentials also needed for closed-loop control. Nevertheless, there are several biomedical applications that require some form of sensing [2]. The most successful and well-known such sensor is the glucose sensor that has helped millions of diabetics worldwide. In recent years, semi-implantable subcutaneous tissue sensors have emerged and have become widely used [12], however, fully implantable glucose-sensing devices are also a commercial reality (see Senseonics Eversense, Germantown, MD, USA) [13]. One common feature of all the aforementioned devices is the fact that they rely on active electronics to perform their complex tasks.

However, there are a multitude of applications, particularly related to sensing, where active electronics are not necessarily needed and where completely passive resonant structures can be exploited to allow sensor interrogation and remote readout. The need for wireless interrogation and communication, as well as biocompatibility, are still present, but packaging and hermeticity can be somewhat relaxed in certain cases. In fact, as will be elaborated further within the paper, depending on the application, packaging, hermeticity and sensor lifetime can be exploited in favor of the application.

This review paper covers a gap in the bibliography, as there does not seem to be another recent review paper focusing exclusively on purely passive implantable sensors. The paper is structured by first providing and discussing some definitions with regards to implantable sensors, passive sensors and their requirements. This is followed by a rigorous discussion of various passive sensors in regards to their sensing modalities, together with their applications. In the last sections, for completeness purposes, material aspects are briefly discussed and a brief overview of communication methods is presented. Finally, a summary and an outlook for future research and developing directions are provided.

### 1.1. What Are Implantable Sensors?

Generally speaking, implantable sensors are devices that are placed within the human body. They can be implanted subcutaneously or deep in the body, in the vicinity of organs or bones. There are devices that are semi-implantable, where only the sensor, in the form of a needle, penetrates the skin, such as Abott’s Freestyle Libre and Dexcom continuous glucose monitoring devices [12]. There are also devices in the form of wearable patches with microneedle arrays for sampling interstitial fluids [14]. However, such devices are not of interest to this review paper and we will be only considering fully-implanted devices. Compared to various wearable devices, implantable devices have several advantages, but also challenges related to their implementation and operation. Some of the advantages include the following:

- Implantable devices can be placed in close proximity to the source of a physiological event, organ, etc. of interest. In contrast, wearable sensors will be placed further away, possibly too far to monitor and detect an event of interest, or pinpoint the source of a signal.
- Implantable devices can be used unintrusively and unobstructively once implanted. In contrast, wearable devices have a certain form factor, can be visible when worn, and may hinder to some extent various activities and movements, while some can have socio/psychological impact to the users and can form the basis for discrimination or bullying.
- Depending on the device, the application and its implantation, an implantable device can be less sensitive to motion artifacts than a wearable device that is in constant contact and friction with the skin.

Challenges related to the implementation, operation and use of implantable devices include the following:

- A wearable device can be easily worn on the body if in the form of a gadget or accessory, or it can be attached to the body if in the form of a patch. However, an implantable device requires a small incision for subcutaneous implantation (which can also be performed with the use of an injector applicator), or needs a more complex surgical procedure for deep implantation.
- Both wearable and implantable devices must be safe and biocompatible. However, the safety and biocompatibility requirements are much more challenging to address with implantable devices [15,16]. As will be elaborated further in following sections, there are permanent implants for long-term sensing and there are also temporary implants for short-term sensing, depending on the application. Short-term implants can also be transient devices to avoid the need for a secondary surgical procedure for device removal [17,18,19]. These further diversify the requirements for implantable devices. Safety and biocompatibility are related to both the materials used, but also the voltage/current levels and light intensity, which can also be related to signal frequency, while the operational temperature of the device must also be considered. Biocompatibility is important not only for the device user, but also for the device itself. The foreign body response isolates the implanted device from the rest of the body, creating an unnatural environment for the sensors, thus leading to erroneous sensor results [20,21].
- Packaging requirements are more challenging in implantable devices. Hermeticity is crucial and specialized accelerated lifetime testing is required to evaluate packaging hermeticity, while sensors can also be co-integrated within the package to monitor package degradation [22,23,24,25,26].
- A mechanism is required to interrogate and communicate with the implantable device. Wearable devices can be easily interrogated using wired (e.g., USB) or wireless (e.g., Bluetooth low-energy, BLE) approaches that are widely used. However, this is not possible with implantable devices, either since it is not possible to connect with a wire or because standard telecommunications protocols are too power-consuming for an implantable device. Several approaches have been implemented both commercially and in the academic literature to address this need, primarily based on inductive or ultrasonic telemetry, while galvanic conduction, radio frequency (RF) and optical approaches have also been proposed [27,28,29,30,31,32,33,34,35,36].
- Depending on the application (long-term vs. short-term, size and power requirements) implantable devices can be either battery-powered or batteryless. Communication and powering can be interconnected, since the carrier signal used for the wireless communication can also be exploited to power the device [27,28,29,30,31,32,33,34,35,36]. Several long-term devices, such as cardiac pacemakers that do not operate continuously, are battery powered. Other devices, such as cochlear implants, are batteryless and require an external device placed in close proximity and aligned with the implant, to power it.

The requirement for battery and wireless power transmission is related to the use of intricate active electronics required to perform complex functions, such as multiplexing, data processing, stimulation, signal recording, digitization, etc. However, particularly for sensing applications, there is the possibility of avoiding the use of active electronics and exploiting the use of passive elements only. Such devices are the focus of this paper.

### 1.2. Active vs. Passive Implantable Sensors and Their Definition

First, a definition is required to discriminate between active and passive devices. Within the context of this paper, we will define a fully passive device as a device that has no active electronics requiring a constant DC power supply. Thus, the review will not consider any examples that use a transistor, either as a discrete device or as part of larger circuit blocks, such as amplifiers or multiplexers. Nevertheless, a passive implanted device must still be interrogated. This is achieved with the use of an external device that transmits a signal. The use of active electronics comes with several advantages, when compared with fully passive implementations. These include the following:

- Several sensor types and sensing techniques require the use of signals and biasing. For example, bioimpedance sensors, where an AC current is injected and the resulting voltage across electrodes is measured over several frequencies, and electrochemical sensors employing voltammetry or amperometry, where either voltage ramps or DC voltages are applied and the resulting current is measured. These sensing approaches cannot be exploited using fully passive approaches.
- The same circuitry can be used to interface to different sensors through multiplexing.
- The use of active electronics allows the implementation of advanced circuit topologies for the recording of very small signals close to the noise floor in the presence of large common-mode signals. This has a direct impact on the overall sensing system’s limit of detection.
- When battery-operated, an active implanted device may not need an external device, being capable of performing all of its operations on its own.
- Active electronics allow on-node processing of sensor data, either in the analog or digital domains, to decide which data to send to avoid transmission of large amounts of data, machine learning deployments, compressive sensing and other signal processing approaches.
- Devices with active components can provide signal digitization and modulation for signal transmission and they can support high data rates, as opposed to fully passive implementations that are analog real-time sensing systems.
- Health-related information is sensitive personal information. Wearable and implantable medical devices can be prone to malicious attacks and unwarranted access to this information [37,38]. Active devices allow the use of encoding and encryption of the transmitted information, e.g., by blockchain. The use of such approaches is even more critical for stimulating devices [39,40].

Fully passive implementations also come with certain advantages when compared with active devices.

- Their arrangement and complexity are much simpler. Simple passive circuits with as few as two or three elements can be exploited, where one or more of its elements can act as a transducer.
- Their low component count allows for ultra-high miniaturization. As will be shown later, passive implantable sensors can be made to have areas of less than a few mm^2^ with practically negligible thicknesses since thin film technology can be exploited. In contrast, active implantable sensors with batteries require an active area of at least 1 cm^2^ and with considerable thickness due to the presence of the battery. This is true even considering the latest developments in batteries in the literature [41,42,43]. The small size is critical in medical applications, to minimize discomfort, incision size and reduce the impact of mechanical motion on the device. In addition, as will be discussed later in some detail, there are applications such as monitoring the temperature on the screws of an orthopedic implant or the blood pressure on a stent implant where the available area for sensor placement is significantly smaller than 1 cm^2^. In such cases, fully passive sensors hold a significant advantage over their active batteryless and battery-powered counterparts. As will be discussed, fully passive systems can be composed of only two components, an inductor and a capacitor. Active, batteryless systems will need these two components (if the same operational frequency and sensing element are considered), but also one or more active components for the required instrumentation, digitization, modulation, power management and communication, plus some additional passives. A system-on-chip (SoC) approach can be implemented aiming to have a single active component. Such a component could be designed and manufactured using an advanced deep submicron technology (e.g., 28 nm or even 3 nm finFET) and could add to the overall size just a few mm^2^. For example, the imaging and neuropotential recording chip manufactured in a 0.35 μm technology node of [44] occupied an area of 0.50 × 5.0 mm^2^, the amperometric-sensing chip manufactured in a 0.35 μm technology node of [45] occupied an area of 10 mm^2^, the pressure-sensing chip manufactured in a 180 nm technology node of [46] is 2.25 mm^2^, the impedance spectroscopy chip of [47] is 4 mm^2^, or the piezoresistive pressure sensor SoC of [48] manufactured in a 65 nm technology node which occupies an area of 0.196 mm^2^. Finally, additional area will be occupied due to the need of an interposer or printed circuit board to provide the interconnections between the sensor, active and passive components and the battery.
- Their low component count and simple arrangements allow for a great degree of flexibility in terms of the choice of materials, designs and functionalities.
- Minimizing the component count and simplifying the device architecture also minimizes potential points of failure as well as reliability, lifetime and manufacturing issues related with heterogeneous integration of devices based on different technologies and dimensions (i.e., sensor device, battery, integrated circuit, interposer or printed circuit board and battery).
- Their operational frequency for remote interrogation is not limited by a telecommunications protocol.
- Their short range (mm to cm) and requirement for a specialized external device safeguards sensitive health-related information.
- Their batteryless operation avoids the need for secondary surgical operation for battery replacement. This is especially important in applications where continuous monitoring of a certain marker is required for the duration of a patient’s lifetime. Such an example is the need for continuous glucose monitoring in diabetic patients or the case of orthopedic and cardiovascular implants. The required battery size in active devices will depend on the application (available space), size of the rest of the implant, the power consumption of the active electronics, whether continuous or discrete sensing is required, and the operational lifetime of the device. Even at low power consumption, no implantable battery could sustain the power needed by a continuously working sensor over a period of decades. For example, the average lifespan of a pacemaker battery is about 6–7 years. In another recent example [49] where the impact of the standby mode and internal memory power consumption was emphasized, it was estimated that a custom-optimized SoC for amperometric and temperature sensors would last 2.5 days when using a 5 µAh 1.7 × 2.2 mm^2^ thin-film battery. With regards to some of the aforementioned integrated circuits for implantable sensing applications, the capacitive pressure sensing chip of [46] consumes a power of 7.8 mW, the impedance spectroscopy chip of [47] consumes ~0.5 mW, while the potentiostat of [45] consumes 9.3 mW. Fully passive systems are by definition zero power systems which do not need a power source to function since they have no active components.
- The batteryless and active electronics-free operation simplifies their realization as transient and flexible or stretchable devices [50].
- The interrogating device can also be made small and simple.
- Their simple arrangement when compared to active devices can make them lower-cost devices, as a specialized custom integrated circuit is not required. Packaging requirements to protect the active electronics or sensors for assessing packaging hermeticity may not be as critical.

**Figure 1:**
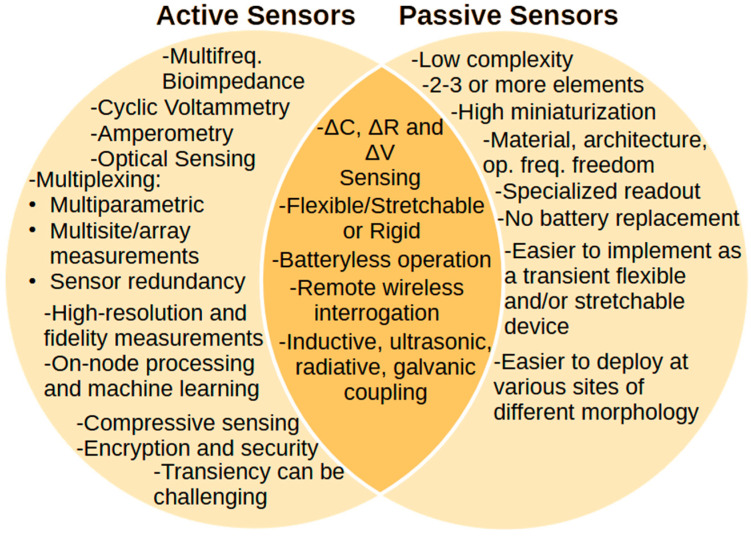
Comparison of active and passive implantable devices.

The above are summarized in Figure 1.

These characteristics make implantable fully passive sensors ideal and indispensable in applications where high miniaturization, simplicity and long-term operation is needed. These include monitoring long-term biomedical implants, such as orthopedic (e.g., spinal cord plates, prosthetic hip or knee replacement devices, implanted screws or tibia fixtures) and cardiovascular devices (e.g., stents) where, for example, strain and pressure or heart rate need to be monitored, or chronic disease monitoring (e.g., diabetes) where various biomolecules (e.g., glucose) and bio-signals need to be monitored over the lifetime of the patient. High miniaturization, simplicity, avoiding the need for re-operation to replace batteries (thus minimizing the potential for infections and increased morbidity and burden to patients) and the use of batteries altogether even if rechargeable due to area constraints, are critical for these applications.

Providing quantitative comparison between fully passive and active (batteryless or battery-powered) devices is not trivial. Their operation, architecture and implementation challenges are very different. There is a lot of freedom in the way fully passive, batteryless and battery-power active systems can be implemented, and their implementation depends on several different specifications. Thus, providing quantitative comparison metrics has the risk of leading to false conclusions.

Based on the above discussions, passive implantable sensors must be purely passive with no active electronic components, they must have no battery or rely on any form of implantable energy storage device, and they must be interrogated remotely and thus wirelessly by a device external to the human body. Finally, they must be fully biocompatible and either be permanent or intentionally transient, and appropriately flexible/stretchable or rigid. In the following section, several examples from the literature are described and are categorized in terms of what they are sensing.

## 2. Passive Implantable Sensors

Due to their very nature, categorizing truly passive implantable sensors is quite challenging. This is due to the fact that the sensing mechanisms exploited in such devices are not entirely independent of their material properties, their communication schemes or their designs. Passive sensors rely on the change of a physical quantity as a response to a specific stimulus. Due to the absence of energy sources and active transmission of their measurements these devices are, in most cases, resonant devices. In general terms, they consist of a resonant structure or circuit such as an antenna or an RLC circuit. An external circuit transmits a signal and records the device resonance. When the device is triggered by the intended stimulus, a physical quantity causes this resonance to change, hence “sensing” the stimulus. This “output” is recorded by the external device and, so, no energy source is needed for the implanted device itself. So, the “communication” elements are also the sensing elements in most cases of passive devices and cannot really be thought of independently. The most commonly used resonant devices are antennas, RF ids, RLC circuits and ultrasound sensors covering frequencies from a few MHz to a few GHz. These frequencies are dictated, in large part, by the properties of the biocompatible materials used, but also by the design specific to each application. So, for example, in the case of an RLC circuit the inductor cannot be made arbitrarily large as it is often limited by the available space at the point of interest within the body itself. On the other hand, the method of communication, as well as its design and frequency, takes into account the depth of the device under the skin, as well as its surrounding environment, to ensure minimal signal loss.

In addition to this, the choice of materials is also part of the sensing scheme in most passive devices. For example, in many applications where the information needed is limited to a relatively short period of time after a surgery, the sensors are designed to be bioresorbable. This ensures that no foreign entity exists in the patient’s body after the required time frame, minimizing possible complications. This transiency is often utilized as part of the sensing mechanism. As an example, an antenna composed of transient bioresorbable materials, and designed to be absorbed at different rates under environments of different concentrations of a substance under investigation, constitutes the sensing element for that substance as well as the communication element for the passive sensor. Similarly, utilizing bioresorbable materials for RLC circuits will also lead to a change in resonant frequency as a response to the materials’ transiency triggered by a designed stimulus.

In other applications, a long-term sensing element is needed due to the long-term effect of the quantity under investigation. Even in the cases where the passive sensors are permanent, though, the choice of materials plays an important role in the sensing strategy of each device. A characteristic example of a material property that defines the sensing strategy of the device is flexibility and stretchability. Sensor circuits designed to use flexible or stretchable materials can react to stimuli, such as strain, pressure or pH, changing their shape, volume, or electrical properties, thus causing a shift in the resonant frequency of the circuit. This frequency shift is a function of the event causing it, thus allowing it to be measured. Moreover, permanent biocompatible materials can be designed to absorb a targeted substance or to react to a physical quantity (e.g., temperature change) causing a change in their volume or dielectric properties. Again, a communication circuit built on such materials will change its resonant frequency.

Intertwined with the material choice and the communication method is the quantity the passive sensors use for the detection of the desired information. A common electrical quantity used, for example, is the capacitance of the sensor. Many sensors are designed to change their capacitance as a response to the target stimulus. A change in a resonant circuit’s capacitance will change its resonant frequency. As explained earlier, there are several ways to achieve this effect in a passive sensor device. A material that absorbs a substance changing its volume or dielectric constant can be used as the dielectric material of the capacitor or as the surrounding material. The electrodes of the capacitor can be made flexible so a change in pressure or strain can lead to a change in the physical distance between them, causing a capacitance change. Microelectromechanical system (MEMS)-type devices are characteristic examples of this scheme. Similar considerations can be made for the inductance of the resonant circuit, often not independently of the capacitance. Other quantities that can be designed to affect the sensing scheme are the reflection properties of electromagnetic waves and the changes in magnetic hysteresis under a stimulus, but such approaches are very rarely used.

It becomes clear that, in order to develop a truly passive implantable sensor, material, communication and method of detection cannot be thought of independently. Certain material aspects such as biocompatibility and, in some cases, transiency are a prerequisite, but depending on the application itself, several different properties might be changeable. It is, for example, possible to develop pressure sensors with similar performances using completely different combinations of materials, communications methods and sensing schemes. It needs to be noted, though, that, in the end, the quality of the sensor can be judged by its specificity, its signal magnitude, its noise floor, its hysteresis and its response with respect to time of operation. These ultimately also define the sensor’s limit of detection (LoD).

**Figure 2:**
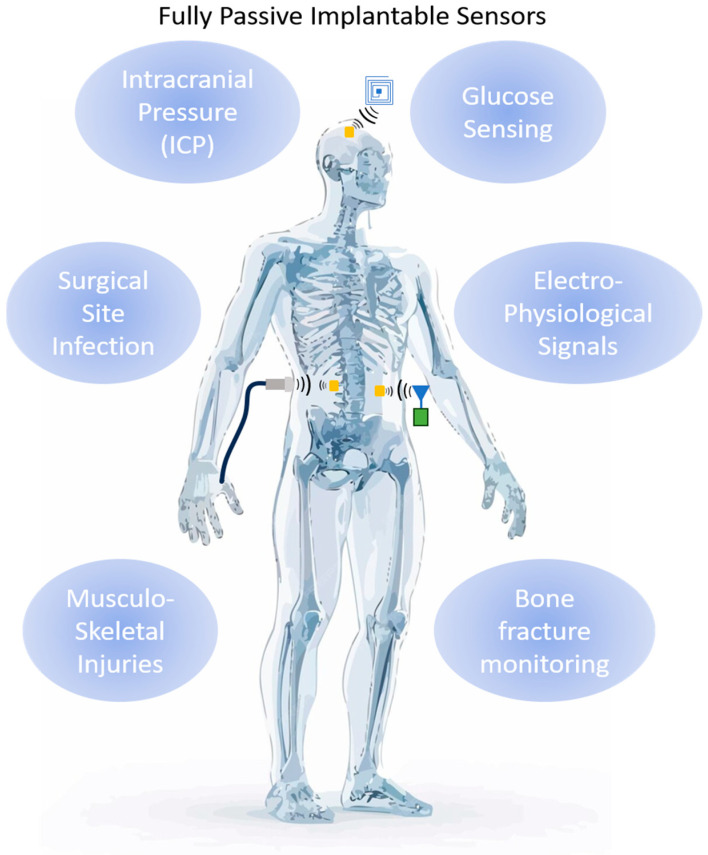
Representative applications of passive implantable sensors. These can be interrogated using ultrasonic (using piezoelectric transducers), inductive (using inductors) or radiative coupling (using antennas). Background human image by @migstc1, from Freepik Company S.L. Malaga, Spain (www.freepik.com, accessed on 22 October 2024).

A potentially more convenient way to categorize passive implantable sensors is by assigning them a specific type, stemming from the physical quantity they are intended to sense. So, there are pressure sensors, strain sensors, pH sensors, temperature sensors, and glucose sensors in the literature. There are, of course, other sensors as well, but they are not as common. Some of them will be discussed in detail below. We note that each sensor in these categories can be used in a variety of medical applications. Figure 2 summarizes several popular applications for passive implantable sensors. Depending on the location of the sensor within the body, the magnitude of the effect and its time scale, the measurement of the same physical quantity can be used as a marker for a different physiological condition. This fact enhances the importance of developing low-cost passive implantable sensors, as their ease of deployment and readout allows them to be used in a wide range of medical applications. In the following section, we present a thorough review of the literature categorized according to the detected quantity. Through the presentation of the various examples, different applications, materials, and methodologies will be described. We note here that the literature on truly passive implantable sensors is quite limited to date, when compared to their active counterparts. An indicative overview of the literature of implantable, wireless, fully passive sensors can be found in Table 1.

### 2.1. Pressure Sensors

The most common type of passive, wireless, implantable sensor in the literature is the pressure sensor. One of the reasons for this is that the measurement of pressure in various locations inside the body offers critical information for a large number of health-threatening medical conditions. For example, pulmonary hypertension is associated with many serious heart and lung conditions [58,59,61,71], intra-abdominal hypertension is associated with high risk of acute Abdominal Compartment Syndrome (ACS) [54], and intracranial pressure is an indicator of severe brain injury [55]. A second reason is that the measurement of pressure can be performed with a variety of techniques that allow the incorporation of many different materials, designs, and communication methods. One sub-category of pressure sensors, and, perhaps, the most common one, is the MEMS-type sensor. In this realization, a solid substrate which includes an air cavity is sealed by a flexible material. A pressure difference on the side of the flexible membrane causes it to deflect, changing the physical separation of the solid and flexible parts of the sensor. This type of device can be either micro- or macro-scale, depending on the application.

Pressure differentials create a change in the physical separation between electrodes and thus cause capacitance changes. This capacitor is integrated as part of an RLC circuit, transforming the capacitance changes into changes of resonance frequency. One of the earliest examples of such an approach was presented in [73]. The pressure-sensitive capacitive sensor was a 6 mm boron-doped silicon diaphragm that formed one of the plates of the capacitor which deflects when pressure is applied to it. The second plate was a metal electrode supported on a glass substrate. The circuit was completed with a gold-electroplated 24-turn planar coil of 1.2 mH. Change in the capacitance changes the resonant frequency of the LC, which varies between 95–103 MHz for a pressure change in the range 0 ± 50 mmHg. This is sensed remotely through inductive coupling. The pressure responsivity and sensitivity of the device were found to be 120 kHz/mmHg and 1579 ppm/mmHg, respectively. The complete sensor occupied an area of 2.6 mm × 1.6 mm.

An example of a fully-transient wireless passive pressure sensor with resonant frequencies around 30 MHz was demonstrated in [56] (Table 1). The sensor used microfabricated MEMS devices composed of Zn/Fe windings and capacitor plates on flexible poly-L-lactide (PLLA) and polycaprolactone (PCL). PLLA was used as the packaging and pressure-sensing material, due to its mechanical properties, and PCL as a bonding and sealing material, due to its low melting temperature. As before, the sensor was an RLC resonator, where the capacitor is pressure-sensitive. Fe is not an ideal choice for high-frequency conductors, as due to the skin effect its resistance becomes prohibitive for achieving a good Q-factor. This can be addressed with the use of Zn that has better properties for high-frequency applications. Zn degrades very slowly under biological conditions. To accelerate this process, the authors galvanically activated the material when electrically connected to iron in saline, making the bilayer structure critical for the accelerated degradation of Zn. The bilayer had a total thickness of 65 μm. The device was manufactured such that it can be folded to create the required 3D structure, after the addition of spacers that define the capacitor air gap between the plates. The device inductance was 1.9 μH below 50 MHz. For the sensor interrogation, a 5-turn, 1 cm diameter coil was used at a distance of 3 mm from the sensor. In air, the baseline resonant frequency of the device was 31.9 MHz, and thus the capacitance was measured to be 13.8 pF. The sensor’s sensitivity was found to be −39 MHz/kPa, which corresponded to ΔC/C_0_ = 5.0% over the pressure range 0–20 kPa. When immersed into saline, these changed to a resonant frequency of 30.5 MHz, a sensitivity of −35 kHz/kPa and a Q-factor that decreased from 20.8 to 18.9. This behavior is commonly reported with RLC sensors. The sensor appeared to stabilize after the first 21 h of immersion and subsequently demonstrated a stable response up to 107 h of immersion; after this point, the sensor behavior clearly deteriorates gradually, and after 168 h, the sensors could barely be interrogated.

A similar, but macroscopic in size, transient pressure sensor with a resonant frequency of 3 GHz was presented in [57] (Table 1). To investigate and compare RF and biodegradation properties of different conductive materials, three different designs were manufactured with pure and alloy metals as well as conductive polymers. The conductive materials examined included Fe, Mg, a magnesium alloy (Mg–2Y–1Zn–0.25Ca–0.15 Mn in wt%), three different iron alloys (Fe–21Mn–0.7C, Fe–21Mn–0.7C–0.5Pd and Fe–21Mn–0.7C–1Pd in wt%) and conductive polymers composed of a biodegradable matrix (polylactide acid, PLLA or poly(-caprolactone), PCL), and conducting polymer nanoparticles, made of polypyrrole PPy (PPy, content 40 wt%) to yield PLLA-PPy and PCL-PPy. Pure Fe degraded slowly in the body. The Fe-alloys exhibited enhanced corrosion rates, based on their Pd content, degrading about twice as fast as pure Fe. However, Mg was the most promising material, since it combined high conductivity (and thus high Q-factor) and promising biodegradation properties. For a faster biodegradation and improved mechanical performance, the Mg-alloy examined was a good alternative. The metal structures were fabricated by electric discharge machining (EDM) from 3 mm-thick plates. A 200 μm-thick PTFE insulating spacer was placed between the capacitor plates to avoid the plates shorting with each other. The polymer-based devices were manufactured via compression molding and laser cutting, or direct compression molding into a mold with laser cutting used only for defining the capacitor gap. The biodegradable conducting polymers PCL–PPy and PLLA–PPy were also good candidates as they allowed devices that are lightweight, while also offering higher flexibility in their fabrication process. They also allowed compatibility with magnetic resonance imaging and transparency to X-rays. The device thickness was again 3 mm and the capacitor gap was 1 mm. Several different device designs were examined with all materials. The metal-based devices were found to have resonant frequency and Q-factor varying between 500 MHz, 660 MHz and 1 GHz, and 8–410, respectively. The polymer-based structures were characterized with inductive coupling which indicated their resonant frequencies to be 2.1 GHz, 2.0 GHz and 3.4 GHz, with Q-factors ranging between 6 and 19.

**Figure 3:**
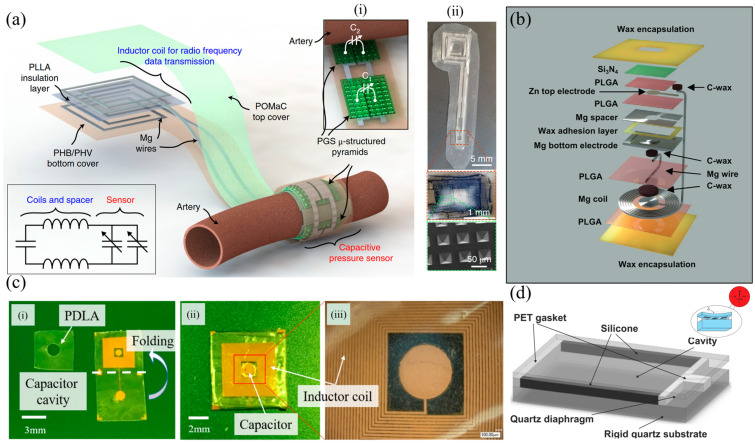
Examples of passive implantable pressure sensors based on the capacitance change caused by a deflection of a membrane. These types of sensors are referred to as MEMS (Micro Electro-Mechanical System)-type in the text. (**a**) The biodegradable and flexible arterial-pulse pressure sensor of [58]. Reproduced with permission from Springer Nature. Published in Nature Biomedical Engineering (https://www.nature.com/natbiomedeng/, accessed on 23 December 2024). (**i**) Close-up illustration of the pressure-sensitive area with the two variable capacitors, before the sensor is wrapped around the artery. (**ii**) The fabricated device and close-ups of the double capacitor sensing region of the device and the pyramid-shaped microstructured sensing layer. (**b**) The biodegradable wireless LC pressure sensor of [60]. Notable is the use of wax and conductive composite wax, among other novelties. © 2020 Wiley-VCH GmbH. Reproduced with permission from John Wiley and Sons. (**c**) The biodegradable PDLA-based wireless LC pressure sensor of [59], formed by folding the device and adding an intermediate insulating spacer that defines the diaphragm. (**i**) Before assembly. (**ii**) after assembly and (**iii**) magnification of the capacitor and inductor of the sensor. Reprinted from Microelectronic Engineering, Vol 206, J. Park, J.-K. Kim, S. A. Park, D.-W. Lee, Biodegradable polymer material based smart stent: Wireless pressure sensor and 3D printed stent, Pages 1–5, Copyright (2019), with permission from Elsevier. (**d**) The SAW resonator-based pressure sensor of [61]. © The Authors 2013. Distributed under the terms and conditions of the Creative Commons Attribution (CC BY) license (https://creativecommons.org/licenses/by/4.0/, accessed on 23 December 2024). No changes were made.

Vessel anastomosis is one of the most critical steps of cardiovascular, vascular and transplantation surgeries. Revascularization is also needed in a range of other procedures, including critical limb ischemia and traumatic vascular repair surgeries, as well as trauma- or cancer-related reconstructive surgeries. Failure of the anastomosis and resulting insufficient blood flow and oxygenation can lead to ischemia and subsequent necrosis and sepsis leading to tissue/graft loss, morbidity, mortality, and the requirement of additional surgical procedures. Currently, sporadic Doppler measurements and visual inspection are used, while the patient is still in the hospital. Continuous monitoring is necessary to improve patient outcomes. An implantable Doppler system is commercially available to address this need, by Cook Medical, the Cook-Swartz probe [74]. However, it is a tethered device. A somewhat different approach, shown in Figure 3a, was presented in [58] to address this need (Table 1). A MEMS-type capacitor was integrated on a flexible substrate which was used to wrap a blood vessel as a cuff, with the bilayer inductor of the circuit placed on a substrate at a distance (not on the blood vessel). Pressure changes within the blood vessel triggered capacitance changes and shifted the resonant frequency of the overall device. This device was designed to be transient because the application of interest was the monitoring of blood flow after surgical vessel anastomosis, so the targeted time frame of its usefulness is limited. Mg was used for the conductors, a 40 μm-thick microstructured poly(glycerol sebacate) (PGS) was used as the capacitor dielectric layer with pyramidal microstructures to increase sensor sensitivity, a 50 μm-thick rigid PLLA spacer was used for the bilayer coils due to its dielectric properties and to make the coil pressure-insensitive, and a 10 μm-thick poly(octamethylene maleate (anhydride) citrate) (POMaC) and a 10 μm-thick polyhydroxybutyrate/polyhydroxyvalerate (PHB/PHV) layer was used as the top and bottom insulating layers of the device, respectively. All materials are extensively used in implantable applications due to their biocompatibility. The very soft and elastic PoMAC was used to interface directly with the artery, while the stiffer PHB/PHV layer would be in contact with the surrounding muscles. PGS has excellent elastic properties in compression mode with negligible viscoelastic behavior and hysteresis. This allowed the device to be more sensitive to artery expansion than respiration. It is clear from these discussions that the material choice was critical and had to be optimized based on the application and the device architecture. The device was designed so that it can also be operated as a fringe-field capacitor, eliminating the need for tight contact between the flexible capacitor substrate and the vessel. This is important as a tightening of the sensor in this position could damage the underlying blood vessel. In the non-contact mode, the device could detect blood flow by the change in the vessel’s diameter as blood pressure changes using the fringe field capacitor as a proximity sensor. Two narrow variable capacitors instead of a single larger one were used. This allowed the sensor to be easily wrapped around arteries with a diameter smaller than 1 mm. The overall device was shown to operate near 500 MHz and was validated in vivo.

The MEMS approach in measuring blood pressure was also used in [59] (Table 1). In this example, shown in Figure 3c, the MEMS pressure sensor was integrated on a polymer stent made of PCL material and designed to be resorbable after a period of time. The sensor was resorbable itself, composed of electrodeposited Cu on poly(D-lactide) (PDLA) and the resonant frequency was 148 MHz, which decreased with blood pressure changes. The incorporation of the MEMS pressure sensor on the stent device allowed for blood pressure measurements inside a blood vessel and at the position of a potential problem, making this device especially interesting. Drug-eluting stents (DES) slowly release pharmaceutical agents and significantly reduce the risk of restenosis; however, once the drug is depleted from the metal stent, patients need to be administered the drug to reduce the incidence of in-stent restenosis. The proposed sensor allowed patients to check the progress of unexpected restenosis themselves and control the dosage of the drug. The sensor used an RLC with an air diaphragm capacitor similar to other examples already discussed, and achieved a sensitivity of 60 kHz/mmHg. The sensor achieved a linear frequency shift within the pressure range of 0–220 mmHg. The device structure and assembly was similar to the one described in [56]. Another MEMS-type diaphragm-based pressure sensor with multiple possible uses in intracranial, intra-abdominal and in pulmonary hypertension, depending on its positioning, was demonstrated in [60] (Table 1). As in the previous examples, the authors designed a transient sensor composed of Mg, PLGA, Si_3_N_4_ and wax (Figure 3b), aiming at applications of short time scales for the detection of post-surgical complications. The device was shown to operate at around 250 MHz, and achieved a sensitivity of 200 kHz/mmHg, a resolution of 1 mmHg and an in vivo operational lifetime of up to 4 days. The flexible electrode of the capacitor was a 2 μm-thick Zn foil embedded in a 10 μm-thick membrane of poly(lactic-co-glycolic acid) (PLGA). The fixed electrode consisted of a 50 μm-deep trench etched into the surface of an Mg foil. Bioresorbable wax was used to attach the bottom electrode and the spacer. Conductive wax (mixture of W powder and wax) was used to electrically connect the coil to the sensor. A freestanding membrane of Si3N4 (2 μm thick) was laminated on the top electrode. Wax (≈500 μm thick) around the perimeter of the sensor, but not on the top electrode, completed the device.

Glaucoma is an eye disease that results in loss of vision and is associated with an accumulation of intraocular fluid, which results in increased Intraocular pressure (IOP). Hence, monitoring IOP is a means of monitoring disease progression. There is a need for continuous and accurate IOP monitoring devices, especially since current methods are inaccurate and unable to regularly monitor the IOP. Spiking events that can be damaging to the optical nerve can thus be missed without prompt clinical action [75]. Two designs were presented in [75], where a variable capacitor or a variable capacitor/inductor structure were realized as the pressure-sensitive components. These were manufactured via micromachining to create passive LC-resonant diaphragm sensors, and achieved sensitivities greater than 7000 ppm/mmHg with a resolution of 1 mmHg. These parylene-based devices were intended for implantation in the pars plana or on the iris of the eye. Animal studies confirmed the device operation and capabilities. A parallel plate capacitor across the diaphragm was implemented as before. This was surrounded by a dual-layer metal inductor. The dual-layer approach increased the inductance. In one approach, the dual-layer inductor was fixed, achieving a resonance at 62 MHz and a responsivity of 0.47 MHz/mmHg. In the second, one of the inductor layers was part of the diaphragm, achieving a resonance at 150 MHz and 1.14 MHz/mmHg responsivity. Thus, with changes in pressure, the intrinsic mutual inductance of the dual-layer inductor would also change, further enhancing the overall pressure sensitivity. The same group demonstrated a similar device in [76]. In contrast to the previous device, the inductor was not supported by the rigid silicon substrate and was thus flexible. A sensitivity of 450 ppm/mmHg and a responsivity of 160 kHz/mmHg were achieved. The device operation was verified in an animal model and measurements could be performed over a distance of 2 cm. These three devices were also summarized in [77].

Within this category, it is essential to highlight the first fully passive RLC diaphragm-based pressure sensor for permanent implantation that was made commercially available, the CardioMEMS [78,79,80]. Currently marketed by Abbott (Abbott Park, IL, USA), the device was initially commercialized by teams from Georgia Tech and MIT in the USA. Intracardiac and pulmonary artery (PA) pressures are the cause of congestion that begins a few days to weeks before the onset of overt symptoms of heart failure (HF) [80]. The CardioMEMS is implanted into the distal PA and measures changes in PA pressure. It consists of a 3D coil and a pressure-sensitive capacitor encased between two fused silica wafers. The assembly is encased in PDMS.

An entirely different approach in the use of the MEMS technology has been demonstrated in [61] (Table 1) and is shown in Figure 3d. In this work, a permanent device was fabricated from a solid and a flexible quartz membrane acting as a diaphragm, separated by two opposing rigid (polyethylene terephthalate—PET) and two opposing soft (PDMS) walls. Al lines were integrated on the flexible quartz membrane creating a Surface Acoustic Wave (SAW) resonator. Generally speaking, a SAW resonator is constructed by depositing metal interdigital transducer (IDT) electrodes and reflecting gratings on a piezoelectric substrate. Among the different options, quartz crystals are a suitable candidate for long-term implantation due to the wide availability of low-cost, high-quality single-crystal quartz wafers that can produce high quality factor (Q-factor) resonators with temperature-insensitive natural frequencies around a desired operating point. A SAW resonator’s resonant frequency depends on several parameters, one of which is the wave propagation velocity, which is dependent on stresses in the substrate. This dependence was exploited for pressure sensing. This device was intended to be placed in the left ventricle (LV) in order to monitor blood pressure in patients who have undergone heart transplant and patients with left-ventricular assist devices (LVAD). A pseudo-normal mode helical antenna was used together with the SAW device. The chosen antenna type provided circular polarization and was thus suitable when the exact orientation of the implant was unknown after implantation. In this approach, pressure differentials resulted in strains within the deflected quartz membrane, shifting the resonant frequency of the device. This sensor was shown to operate at frequencies around 0.87 GHz and was validated in an animal model, demonstrating the first known wireless pressure data recorded from the left ventricle of the heart of a living swine. A pulsed RF signal was used to excite the SAW resonator, with a 100 Hz pulse repetition frequency. The resonator reached its steady state after 1 μs, at which point the RF excitation was turned off. The resonator energy was then radiated back via the implantable antenna in the form of a decaying sine wave at the device’s resonant frequency. This was a 1–2 μs-duration decaying signal, which is the signal of interest. A delay was introduced between the excitation and recording cycles to avoid recording any reflections of the excitation from surrounding objects. This echo signal from the SAW sensor could be recorded with an external device such as the one presented in [81]. The long-term hermeticity of the sealed cavity was highlighted as an issue with the proposed device. The permeability of polymers and elastomers, and in particular PDMS, to gasses and certain liquids leads to sensor drift due to the gas exchange between bloodstream and the sensor cavity. Detailed analysis of the phenomenon, together with appropriate choice of materials, is needed to address this issue. It is important to note that this seminal work progressed over the years to achieve commercialization [82]. Similar developments from the same group have also led to a product for intracranial pressure monitoring [83].

**Figure 4:**
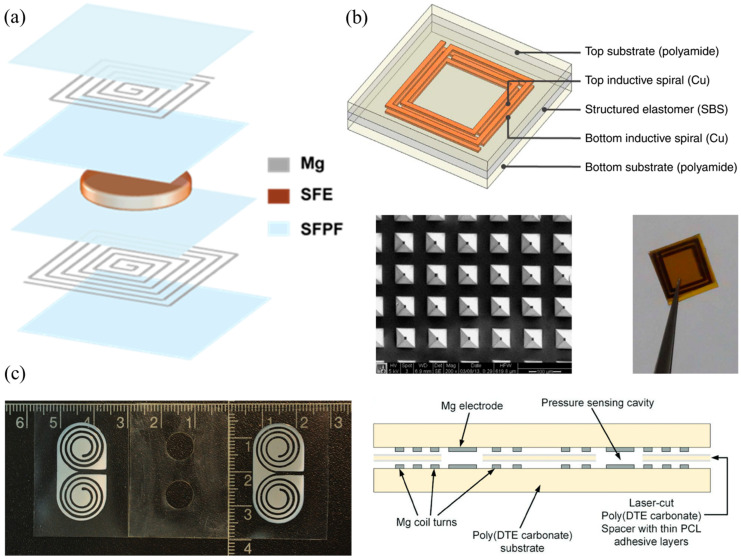
Examples of passive implantable capacitive pressure sensors based on soft, deformable dielectric layers and structured elastomers and diaphragms. (**a**) The degradable LC pressure sensor of [55]. It consists of layers of a composite silk fibroin protein film (SFPF) as the sensor substrate and intermediate dielectric and a hydrogel silk fibroin elastomer as the dielectric layer of the capacitor. Mg is used as the conductor. © 2023 The Authors. Distributed under the terms and conditions of the Creative Commons Attribution (CC BY) license (https://creativecommons.org/licenses/by/4.0/, accessed on 23 December 2024). No changes were made. (**b**) The permanent LC pressure sensor of [84] and the pyramidal-structured capacitor dielectric layer of the device. Reproduced with permission from Springer Nature. Published in Nature Communications (https://www.nature.com/ncomms/, accessed on 23 December 2024). (**c**) The bioresorbable pressure sensor of [85] and its cross-section. Mg is used once again as the conductor. © 2019 WILEY-VCH Verlag GmbH & Co. KGaA, Weinheim. Reproduced with permission from John Wiley and Sons.

A similar approach to the MEMS-type sensors, but omitting the air gap and, thus, simplifying the fabrication process, was followed by the authors of [55] (Table 1) and is shown in Figure 4a. This LC pressure sensor was developed for monitoring the intracranial dynamic pressure which constitutes an important marker for patients with brain trauma. Traumatic brain injury (TBI) raises intracranial pressure by 5–10 mmHg. This may block blood flow and lead to fatal ischemia or it can lead to secondary brain injury. TBI is a major health and socioeconomic problem globally, and untethered solutions for intracranial pressure monitoring are in need. The device was designed to be resorbable, eliminating the need for removal surgery after the patient’s recovery. Two metal coils were integrated on either side of a soft hydrogel elastomer (SFE). Changes in pressure resulted in the deformation of the soft elastomer resulting in changes in the LC resonant frequency. Silk fibroin (SF) is naturally biodegradable and biocompatible. SF samples with different force/chemical properties were prepared through mesoscopic regulation, to achieve controlled and complete degradation and reduce the material’s brittleness and improve its chemical stability. The materials chosen were silk fibroin composite film (SFPF), designed to have good flexibility and stability, which was used as the encapsulation layer and the substrate, and silk-based hydrogel elastomer (SFE), designed to have low modulus and high elasticity, which was used as the dielectric layer, as well as 300 μm-diameter Mg wires for the conductors, and bioglue. The soft device was composed of several layers to create a 3D structure, where coiled Mg wires formed both the inductors and capacitor plates to create a device with a size of 2 cm × 2 cm × 5 mm. The dual coil structure increased the total inductance. Applied pressure decreased the distance between the two inductors, changing the capacitance of the circuit and the resonant frequency of the device. The SF-based all-protein sensor achieved a sensitivity of 4.44 MHz/mmHg for pressures ranging between 0–20 mmHg, and was able to operate with no degradation in performance for 36 h, after which biodegradation began to impact the sensor behavior. Measurements with a distance between sensor and interrogating coil up to 13 mm were presented, and the resonant frequency of the device could be varied within 200 MHz to 450 MHz as a function of the applied pressure.

Another pressure sensing approach (Figure 4b) was presented in [84]. These flexible, passive, millimeter-scale sensors, measuring as small as 1 × 1 × 0.1 mm^3^, were able to capture a human heart rate in real time. Devices sized 2.5 × 2.5 × 0.1 mm^3^ were implanted in mice to monitor real-time in vivo ICP. Increasing spiral length reduced device resonant frequency. Scaling down device area or number of turns increased the sensor resonant frequency. Sensor arrays were also demonstrated that allowed concurrent spatial pressure mapping. Sensors of size 4 × 4 mm^2^ could be interrogated in air over a distance of 15 mm from the readout antenna, while 1 × 1 mm^2^ sensors could be interrogated over 3 mm. The device consisted of a deformable dielectric layer sandwiched between two inductive spirals. Under applied pressure, the vertical distance in the 3D structure between the two spirals was reduced. This increased the effective coupling capacitance, shifting the resonant frequency lower. The inductive structures were fabricated on polyimide. The pressure-sensitive dielectric layer was realized with a microstructured styrene-butadiene-styrene (SBS) elastomer. SBS has more favorable (lower loss) characteristics for high-frequency applications when compared to poly(dimethyl siloxane) (PDMS) or polyurethane. The pyramidal microstructured elastomer allowed the device to be more sensitive than when it is unstructured. This is due to the reduced viscoelastic behavior of thin films and the change in separation distance, as well as the change in effective permittivity, since as the pyramidal elastomer microstructures deform, they fill the air gaps between them. The devices demonstrated a linear response between 0–100 mmHg. Beyond 100 mmHg, the sensitivity of the device was reduced, as the microstructures of the insulating film were fully deformed. The authors also presented an approach to interrogate such sensors using a benchtop network analyzer, designed to overcome the operating frequency limits of traditional passive strategies, allowing high frequency measurement irrespective of the interrogating antenna design and close to its self-resonance frequency.

Figure 4c shows the RLC-based sensor presented in [85]. It was fabricated through the electron-beam evaporation of Mg through 3D-printed shadow masks onto 430 μm compression-molded poly (DTE carbonate) substrates to form 7 μm conductive tracks. Spacers with a thickness of 65 μm and 95 μm were two substrates with patterned conductive features to create four coils and two varying capacitors. Poly (DTE carbonate) degrades hydrolytically and resorbs in the body. The sensors were interrogated at distances between 4 mm and 10 mm, were readable up to 14 mm and achieved a sensitivity of −6.0 kHz mmHg^−1^ within a linear range up to 200 mmHg that is suitable for detecting physiologically relevant pressures. Non-idealities in the manufacturing process led the resonant frequency of the manufactured devices to vary between 76.96 and 94.20 MHz. The possible distance between coils can be increased by increasing the conductivity of the coils. The authors reported that alignment between the two coils (sensor and reading coil) affected the detected resonance frequency. The largest errors took place when the coupling between the coils was the strongest, thus at the closest distance. A displacement of 1 mm in the *X*- or *Y*-direction altered the measured resonance frequency by 6 kHz, which translated to 1 mmHg, while a displacement of 2 mm in the *X*-direction or 3 mm in the *Y*-direction altered it by 34 kHz. When immersed into saline, the sensor started drifting within the first 12 h.

Finally, a completely different sensing scheme for pressure sensing without the use of any electrical device was presented in [54] (Table 1). In this work, the authors developed a permanent sensor for monitoring intra-abdominal pressure for long-term detection of intra-abdominal hypertension (IAH). Patients with an intra-abdominal pressure >2.7 kPa are at high risk for developing either acute or subacute abdominal compartment syndrome (ACS). IAH reduces blood flow to abdominal organs, leading to multi-organ dysfunction. This may include pulmonary, cardiovascular, renal and central nervous system dysfunction. ACS can lead to mortality, with a rate of 80% to 100% if diagnosis and treatment are delayed. Current recommendations for high-risk patients involve measurement of intra-abdominal pressure every 4–6 h and as frequently as every one hour, once organ dysfunction evolves, until conditions have improved. The gold standard involves the insertion of a balloon-tipped catheter into intra-abdominal organs; a highly invasive technique. Thus, a simple method allowing frequent measurement of low or no invasiveness is needed. The device consisted of a PDMS microchannel (8 mm in length, 0.45 mm in width, and 0.45 mm in height) with a reservoir at one end, sealed by a PDMS membrane. The reservoir (1.6 mm × 1.6 mm × 0.8 mm) was filled with water, which remained therein due to surface tension and did not enter the microchannel. The reservoir was made slightly larger than the micro-channel to provide sufficient water to fill the entire channel under pressure. The top PDMS layer was 1.3 mm thick. Pressure differentials on top of the reservoir resulted in the water moving in the microchannel at lengths analogous to the pressure applied. The device could be “read” using a 40 MHz medical ultrasound imaging system (21–55 MHz transducer). The sensor demonstrated experimentally a linear sensitivity of 42 kPa/mm and a spatial resolution of 1.2 kPa/30 μm, in the physiological range of ACS. The advantage of using ultrasound is that the implanted device can be implanted deeper into the tissue. Having smaller wavelengths with less attenuation in biological tissues, ultrasonic waves are more efficient for energy transmission, while due to the smaller size of ultrasonic transducers, the implanted devices can be smaller. In addition, ultrasonic imaging systems are widely available in clinics and hospitals. Cell culture studies confirmed both the biocompatibility and leak-proof properties of the device.

Another non-electrical pressure-sensing method was presented in [86]. This passive sensor relied on near-infrared (NIR) excitation, which is minimally absorbed by tissue and is not visually perceivable. The optical cavity of the sensor reflected a pressure-dependent resonance signature when excited by NIR. This is IOP-dependent. The sensor achieved an average accuracy of 0.29 mm Hg in the range 0–40 mm Hg. The sensors were implanted in animal models, allowing continuous monitoring of IOP over a period of 4.5 months at a rate of 10 Hz. The measurement of transient short-term variations was also possible. The authors argued that a major limitation of LC-based sensors for IOP monitoring is the overall size of the implant due to the size of the inductor, the size of the external readout coil, the complex instrumentation and the short interrogation distances possible. The proposed device, on the other hand, was 900 μm diameter and 600 μm high, allowing its use with silicone haptics or intraocular lenses. The device could be interrogated over a distance of 3–5 cm, which could be extended to 10 cm according to the authors. The sensor consisted of two halves. The top half was a micromachined silicon ring with an SiN diaphragm. The bottom half was a solid reflective silicon surface that acts as a mirror. The 7 μm gap between the diaphragm and the mirror formed an optical resonant cavity, which was further enhanced by the deposition of a gold nanodot array onto the membrane. This matched the reflectivity of the diaphragm with the mirror and maximized the amplitude of the optical resonance. A 200 × 200 μm^2^ array with a dot diameter of 600 nm and dot-to-dot pitch of 1 μm was used. This doubled the amplitude of the cavity resonance [86]. Through the use of an artificial neural network, the authors achieved improved sensing performance [87]. A similar interferometric sensing approach was presented in [88]. A 200 nm silicon nitride diaphragm with a 10 μm SU-8 spacer, and a 200 μm glass substrate were used to form the sensor. Interference fringes between the diaphragm and the substrate formed as a function of the applied pressure when a monochromatic light source was directed towards the cavity. An accuracy between ±0.3 mmH and ±0.6 mmHg could be achieved within a range of 0–60 mmHg. The device measured 1.5 mm × 1.5 mm × 0.4 mm. The incident light needs to be normal to the device as different angles could result in erroneous measurement.

### 2.2. Strain Sensors

**Figure 5:**
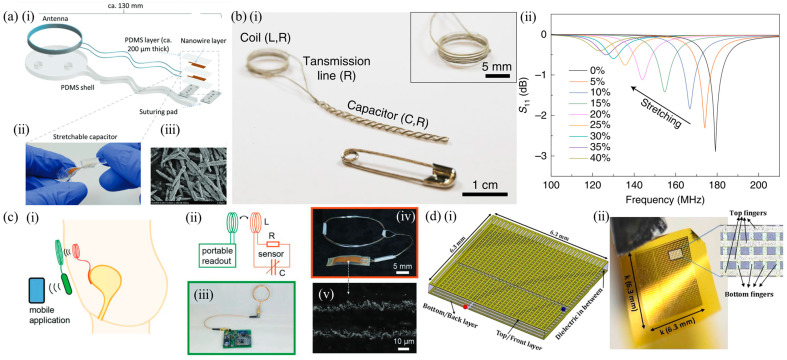
Characteristic examples of flexible and stretchable passive, implantable strain sensors. (**a**) (**i**) Illustration of the architecture of the LC strain sensor for musculoskeletal applications proposed in [64], (**ii**) the fabricated device being twisted and (**iii**) the Au-TiO_2_ nanowires used to form the capacitive sensor plates. © 2023 The Authors. Distributed under the terms and conditions of the Creative Commons Attribution-Non Commercial-No Derivs License (CC BY-NC-ND 4.0) (https://creativecommons.org/licenses/by-nc-nd/4.0/, accessed on 23 December 2024). No changes were made. (**b**) (**i**) The LC strain sensor of [92]. It consists of helical electrodes to implement a parallel plate capacitor with an air gap between plates that was also exploited to aid the suturing of the device in connective tissue. (**ii**) Measurement spectra of S_11_ for applied tensile strains up to 40%. Large resonant frequency shifts were achieved with the proposed device. Reproduced with permission from Springer Nature. Published in Nature Electronics (https://www.nature.com/natelectron/, accessed on 23 December 2024). (**c**) A similar LC strain-sensing device by the same group targeting bladder volume monitoring [93]. Illustrations of the (**i**) use of the device and (**ii**) its operational principle, (**iii**) the external interrogating device, (**iv**) the implantable device and (**v**) the pressure-sensing parallel plate capacitor. © 2018 WILEY-VCH Verlag GmbH & Co. KGaA, Weinheim. Reproduced with permission from John Wiley and Sons. (**d**) The metamaterial-based flexible permanent strain-sensing device developed for orthopedic applications with a nested split ring resonator topology [94]. (**i**) Illustration of the device architecture and geometry. (**ii**) The fabricated device. Inset: Close-up of the top and bottom fingers of the device. Reprinted from Sensors and Actuators A: Physical, Vol 255, A. Alipour, E. Unal, S. Gokyar, H. V. Demir, Development of a distance-independent wireless passive RF resonator sensor and a new telemetric measurement technique for wireless strain monitoring, Pages 87–93, Copyright (2017), with permission from Elsevier.

Next to pressure sensors, strain sensors are the second most discussed category in the literature. Strain is an important parameter in several medical applications ranging from cardiomyocyte detection to orthopedic implants, to musculoskeletal soft tissue damage detection and bone-healing monitoring. Orthopedic applications represent an important family of applications where implantable sensors, and especially fully passive devices, can have a tremendous impact, with several such examples in the literature, as summarized in [89,90]. In the case of strain, the flexibility of the materials used and the device fabricated is most often utilized to transform the physical quantity of investigation to a measurable quantity. For example, a stretchable strain sensor system for wireless measurement of musculoskeletal soft tissue (MST) strains was demonstrated in [64] (Table 1) and is shown in Figure 5a. MSTs (e.g., ligaments and tendons) are crucial for stabilizing joints, providing joint proprioception and absorbing external impacts. MST injuries can thus limit musculoskeletal function, while they can also initiate tendinopathy and osteoarthritis, further impacting quality of life. Assessment of MST strains in vivo (e.g., using ultrasound) has been problematic thus far, motivating the development of alternatives. The proposed sensor was designed to be a permanent implant for long-term strain monitoring. An RLC circuit was fabricated, with the capacitor being stretchable. The capacitance value depended on the strain imposed on it, and so the resonant frequency of the device changed. When compared to resistive strain sensors, capacitive strain sensors exhibit lower hysteresis, less overshoot and higher linearity. The capacitive sensor was composed of two conductive plates made with biocompatible gold titanium dioxide nanowires (Au-TiO_2_ NW), separated by a layer of dielectric silicone rubber, resembling a membranelike “sandwich” plate structure that is embedded in biocompatible polydimethylsiloxane (PDMS). The inductor was implemented simply by a wire loop. This device was demonstrated to operate between 11.5 MHz and 15 MHz depending on the applied stress. It demonstrated a linear behavior between 0 and 25% strain, whereas macroscopic MST failure takes place between 8 to 15% strain. Following 100,000 cycles of fatigue loadings, it remained electronically stable for one week. It exhibited a resolution of 0.1% strain, which is equivalent to about 9 μm. Sensor interrogation was achieved by transiting a continuous sine wave from the transmitting coil of the external device to the implanted coil using near-field magnetic coupling. In this way, the LCR sensor is energized in a forced oscillation, at the signal frequency. After turning off the transmitted signal, the exponentially decaying oscillation of the sensor signal could be measured through the external coil. The frequency of this signal, however, changed from the forced value to the self-resonance frequency of the RLC circuit. Simultaneous interrogation of several sensors was possible by synchronizing different readouts appropriately. To avoid signal interference, a minimum distance of 10 cm between implanted coils was required. Due to readout system limitations, a sensor Q-factor above 10 was required. Consequently, high conductivity and low resistive piezo-sensitivity were required to ensure the Q-factor would not decay due to a strain-induced increase of electrode resistance. The sheet resistance of Au-TiO_2_ NW electrodes was below 10 Ω□^−1^ at strains of up to 100%. This allowed a Q-factor of 10 or greater to be achieved at strains slightly above 25% strain.

A similar approach was followed in [91] for monitoring the growth and strain response of bone in osseointegrated prostheses. Determining whether sufficient osseointegration has occurred is essential for allowing loading of the device. In addition, bone can develop fractures due to excessive load on the prosthesis. Such studies offer the potential of gaining greater insights in orthopedic implant osseointegration, providing the possibility of monitoring postoperative complications and bone healing, but also long-term implant performance and implant lifetime. They also allow the advancement and optimization of osseointegrated mechanical components, the surgical procedures required, and the post-operative rehabilitation phase. Thus, morbidity and overall patient outcomes can greatly improve. The device was manufactured using standard thin-film microfabrication techniques to pattern Cr, Ti and Au on a flexible 25.4 μm-thick polyimide layer sandwiched between two 35 μm-thick layers of copper. The realized conformable wireless device allowed the device to be attached on the curved surface of the orthopedic implant and was composed of two RLC-based circuits, each with a different resonant frequency and different functionality. The first circuit was composed of a spiral inductor (1.386 mH with 0.934 Ω) and a 7.7 mm × 7.7 mm parallel-plate capacitor (178.48 pF) fabricated from metal electrodes and a 10 μm polyimide dielectric layer. The dielectric layer changed thickness due to planar strain. Its purpose was to monitor axial strain in the bone. This circuit achieved a resonant frequency of 10.1 MHz. The second circuit included the same inductor, but a differently sized capacitor (5.55 mm × 5.55 mm) to achieve a different capacitance (92.73 pF) and thus a different resonant frequency (14 MHz). This circuit also included a highly nonlinear titanium resistive element with an initial value of 15 Ω acting as a fuse. Its purpose was to monitor hoop strain associated with circumferential bone growth common in compress osseointegrated prosthesis fixtures. The resistance of this element exponentially increased under monotonic increasing hoop strain. Eventually, the fuse ruptured at the designed threshold strain, at which point the second resonant peak disappeared. An external 5 μH coil with an impedance analyzer was used to experimentally validate the device operation. The first RLC circuit demonstrated a linear relationship between its resonant frequency and low levels of strain, with a sensitivity of 4.555 Hz per unit microstrain.

In an example from 2021, a wireless fiber strain-sensing system was proposed that combined a suturable capacitive sensor with an inductive coil for the real-time monitoring of physiological strains in MST connective tissue [92]. The strain sensor was formed of two fibers in a double helical structure (3 turns/cm), as shown in Figure 5b, with a hollow core (500 μm diameter) and had a sensitivity of ΔC/C_0_ of ~12 for strains of 15–27.5%. The suturing process would typically strain the sensor towards the 15% range. Ligaments and tendons can typically be stretched up to about 10% before they rupture. The highly conductive and stretchable fibers were realized by creating composites through the absorption of Ag^+^ ions into PU-based commercial stretchable fibers. These ions were subsequently chemically reduced into metallic Ag nanoparticles. When strained, the fibers are gradually straightened out and their distance gradually diminishes, increasing the capacitance between them. The operation of the sensor could be split into two operational modes. Within the first mode, the conductive fibers in the sensor were straightened out rather than intrinsically stretched, which is what took place in the second operational mode. Thus, the actual resistance of the composite fibers changed only slightly and the Q-factor of the device was not altered significantly. This led to reduced hysteresis. A network analyzer with an external interrogating coil was used and the reflection coefficient S_11_ of the circuit was monitored. The resonant frequency of the wireless system decreased with increasing tensile strain as the capacitance increased from 180 MHz to 120 MHz for strain between 0 and 40%. A frequency shift of the order of tens of megahertz was obtained, with high and sharp peaks, due to a high Q-factor. A magnet was also included with the coil, to allow alignment with the external coil when the sensor is implanted. The hollow structure of the sensor also allowed the device to be fabricated directly onto a medical suturing thread. The same group also demonstrated a similar passive sensor, shown in Figure 5c, for monitoring the volume of the bladder for patients with neurogenic lower urinary tract dysfunction and patients with spinal cord injury [93]. A soft stretchable conductor was proposed, which was based on gold-coated titanium dioxide nanowire (Au-TiO_2_NW) layers embedded in an ultrasoft silicone elastomer (Dragonskin, Smooth On, USA). This conductor had the ability to remain conductive at strains as high as 100% without substantial cyclic fatigue. The material achieved a sheet resistance of 0.54 Ω□^−1^ and a conductivity of 4630 S/cm for an average layer thickness of 4 µm. The capacitance of the planar plate capacitor increased upon strain due to geometrical changes. This, together with the co-integrated inductor, led to resonant frequency changes between 1 and 30 MHz.

Several works involving the group of V. Demir have been published, demonstrating fully passive metamaterial-based devices for orthopedic applications [65,94,95,96,97,98,99,100,101,102] (Table 1). This body of work focuses on the development of strain-sensing RF passive structures for monitoring the healing of bone fractures. Of particular importance are long bone fractures. About 10% of all bone fractures do not heal properly. This is primarily due to improper load distribution and strain profiles during the healing process. Stainless steel or titanium plates are often used in severe fractures which bear most of the load during the early phases, with the load gradually transferred to the tissue as healing progresses and as it ossifies. The first 30 days are crucial and monitoring is essential to determine early if the healing is normal or aberrant [98]. The applied load to the stainless steel plate deforms it and modifies the resonant frequency of the device as the capacitance of the film between the metal and the substrate changes. The device consisted of a two-turn coil structure with spiral geometry to implement a distributed inductor and capacitor on a highly resistive Si substrate with an Si_3_N_4_ dielectric layer occupying an area of 270 μm × 270 μm. The baseline resonant frequency was at 13.71 GHz, with a Q-factor of 38, achieving a sensitivity in the region of 0.0918 MHz/N. Three such elements were fabricated side by side, to demonstrate a telemetric system, with two acting as antennas and the middle one as the sensing element. Similar circular topologies were demonstrated in [99], which achieved larger frequency shifts and sensitivities than rectangular alternatives. Compared to conventional RF structures, metamaterial-based implementations achieve higher Q-factors (and thus better signal-to-noise ratios) and larger resonant dips. A flexible device of this type manufactured on a polyimide substrate was presented in [95]. A 100 nm Au layer was first deposited followed by deposition of a 100 nm thick Si_3_N_4_ dielectric layer and a subsequent 100 nm Au layer. The bottom Au layer was required due to the polyimide substrate, and its role was to enhance the dip at the resonant frequency, while also creating a strain-sensitive parallel plate capacitor. A split ring resonator was patterned with a 2.22 mm outer length and a 1.38 mm inner length, a 140 μm inner width and a 140 μm outer width, with a 280 μm inner spacing and a 280 μm outer spacing. A 5 × 5 array was created leading to an overall device size of 1.5 × 1.5 cm^2^. The operational frequency of the sensor was at 12.208 GHz and the device achieved a sensitivity of 0.292 MHz/kgf, corresponding to 13.83 × 10^−3^ MHz/microstrain. When compared with similar devices on a silicon substrate [96,97], the polyimide-based devices achieved greater sensitivity and linearity [95]. Several split-ring resonator architectures were presented in [65] and a nested approach was proposed which reduced the operational frequency of the device and achieved a sensitivity of 1.09 kHz/kgf. It was composed of Au, Si_3_N_4_ and Si materials. The sensor was, in essence, a comb-like structure. The application of strain caused a deflection of the device substrate and elicited a capacitance change, thus changing its resonant frequency. Overall, the system was shown to operate around 530 MHz. Similar comb-like devices with resonant frequencies in the 44.3 MHz range were attached to fracture fixation plate implants using an epoxy and, prior to implantation, were coated with PU [101]. They were implanted in union and non-union models in in vivo animal experiments. In another example, the authors attached an array of five similar comb-like structures manufactured on a flexible polyimide substrate onto an intramedullary nail. Each sensing element was square with 8 mm sides and 0.8 mm thickness. A multi-antenna array of five evenly spaced antennas was developed that allowed simultaneous interrogation of five sensors. Each antenna emitted at a unique frequency inducing a resonance within each sensor, which depended upon its architectural features. The antenna array was optimized to reduce cross talk. In the above studies, misalignment and variation in the distance between sensor and interrogating antenna caused inaccuracies. To address this issue, a distance-independent passive RF resonator sensor and a new telemetric measurement methodology were proposed in [94]. Comb-shaped split-ring structures, shown in Figure 5d, were patterned on both sides of the flexible dielectric substrate to form a distributed LC tank circuit. The structures were aligned by 90° rotation, with both layers contributing to the sensitivity of the device. A high Q-factor allows precise identification of the resonant frequency, a strong coupling between the two antennas, a higher SNR and thus a longer interrogation distance. One approach to improve the Q-factor is to increase the device metal’s thickness to be two times thicker than the electrical skin depth [94]. To address this requirement, an Au thickness of 6 μm was used.

In [62] (Table 1), the authors developed a flexible magnetic material, namely a gelatin methacrylate (GelMA)/Fe3O4 magnetic hydrogel film. This permanent material was so flexible that it allowed its incorporation onto cardiac tissue. In the presence of strain, the material deformed and changed its relative magnetic permeability. This was wirelessly detected by a vibrating sample magnetometer which measured magnetic hysteresis loops. This device was designed for cardiomyocyte detection. Another magnetic material-based approach employed the use of magnetoelastic materials [103,104,105,106]. As described in [103], when exposed to AC magnetic fields, resonance was achieved. This is a strain-sensitive parameter. A low-frequency (e.g., 1 kHz) AC field was applied to magnetize the device with the use of a coil. The magnetized device generated a secondary magnetic field that is strain-sensitive at a much higher frequency (e.g., 100 kHz) than the exciting signal and was measured by a secondary external device. This secondary coil was placed perpendicularly to the other coil to avoid measuring the field generated by it. Thus, this was another entirely passive and wireless sensing approach, which, however, requires two coils, while the implanted device was composed only of the magnetoelastic material. One such material was Fe_40_Ni_39_Mo_4_B_18_ (2826 MB, Metglas Inc., Hong Kong, China). The interested reader is referred to [105] for more in-depth discussions in the theory of magnetoelastic materials. Metglas was also used in [107], where only a recording coil was used to interrogate the magnetoelastic device. Stochastic noise filtering was implemented to aid in sensor signal interpretation and decision-making. For long-term safe, biocompatible implantation of such materials, attention is required to the quality and lifetime of the packaging materials, as common ferromagnetic materials (Co and Ni) are cytotoxic. Nevertheless, biocompatible and biodegradable magnetoelastic materials have been demonstrated, e.g., iron-gallium degradation products [108]. The exciting magnetic field causes the device to vibrate, leading to vibration-controlled degradation. This approach has been exploited for monitoring artificial bone implantation, where the sensor was co-fabricated and integrated within the artificial bone, or for monitoring long-term fracture healing, for example together with the use of internal plates used to support a broken bone as it is healing. During the healing process (artificial) bones are subject to axial load, bending, and torsion. Non-union due to bone loss, secondary bone fracture or improper healing are common complications related to the use of internal plates, while chronic overload can damage and reduce the lifetime of artificial bones. To improve outcomes and reduce morbidity, it is essential to frequently assess the healing process to thus allow informed decisions and timely interventions. However, fracture healing monitoring is typically achieved through X-ray imaging, which is unsafe for frequent use, while interpretation of the images may be subjective in certain cases, as is manual palpation.

Finally, as in the case of the pressure sensors, a sensing scheme for measuring strain in orthopedic implants using a microchannel and a reservoir of water was developed in [63] (Table 1). This device was designed to be a permanent implant and was composed of PMMA. A spiral microchannel was fabricated with a reservoir of water at one end, on a flexible substrate. The application of strain induced a movement of the water in the reservoir into the microchannel, which is otherwise empty, due to surface tension. The length of the water leakage into the microchannel could be related to the amount of strain applied. So, by monitoring the position of the water by a commercial medical ultrasonic device, the strain in the position of the implanted device could be measured.

### 2.3. pH Sensors

Monitoring of surgical sites for complications following surgery can have a significant impact on post-surgical patient management [109]. It can allow timely intervention, reducing the need for secondary operations and revision surgery and thus reduce morbidity and mortality. The complications that can arise post-surgically are primarily surgical site infection (SSI) and tissue ischemia. A particular application of interest is intestinal anastomosis, the failure of which can be devastating to patients. Passive sensors can be implanted in the vicinity of the surgical site and detect SSIs and tissue ischemia early. To avoid reoperation, the device should be transient, however intraluminal placement can allow the use of non-transient devices, which can leave the body naturally when they are no longer needed. pH is a marker of infection, ischemia and inflammation [90,110,111]. Continuous monitoring of inflammation markers is especially advantageous following surgical procedures [51,52]. In addition, pH levels constitute a physiological marker for other conditions, such as gastric and intestinal anastomotic leakage, early in the postoperative course before the development of clinical issues [53]. Injury, infection and ischemia cause the tissue to become acidic (local acidosis), leading to measurable reduction of pH. This arises mainly by the increase of lactic acid production as a result of anaerobic metabolism of infiltrated white cells (neutrophils), and due to the production of fatty acid byproducts of bacterial metabolism. The intracellular pH of an activated neutrophil is very low. This highly acidic content is released when neutrophils die, further reducing the pH. Device materials can be made transient as a function of pH. One example of a passive implantable pH sensor was presented in [51] (Table 1), where the biodegradation process of the device itself was exploited for sensing. The fabricated slot antenna was composed of Mg and it measured 0.5 cm in width and 2.5 cm in length. Two narrow bridges connected square pads that, due to their smaller size, degrade quicker than the wider main structure. Thus, degradation of these two bridges breaks up the antenna into three independent structures. The initial resonant frequency was found to lie between 1.4 GHz and 1.6 GHz [51]. This antenna was designed to degrade at different rates when in the presence of different pH levels. The degradation of the antenna occurs in a controllable fashion leading to a measurable shift of the device resonant frequency. A very similar device which also utilizes the speed of biodegradation in different pH level environments was presented in [52] (Table 1), as a means of providing soft-tissue trauma monitoring and infection detection. This frequency selective surface (FSS) sensor was an RFid tag composed of a cross made of silver paint on a transient polylactic acid (PLA) substrate, 1.3 cm in length. The cross was designed to have break points, so that exposure to higher pH levels promoted the breakage of those points initially. This dramatically shifted the resonant frequency of the RFid tag, leading to pH detection. The device was shown to operate between 4 GHz and 11 GHz.

**Figure 6:**
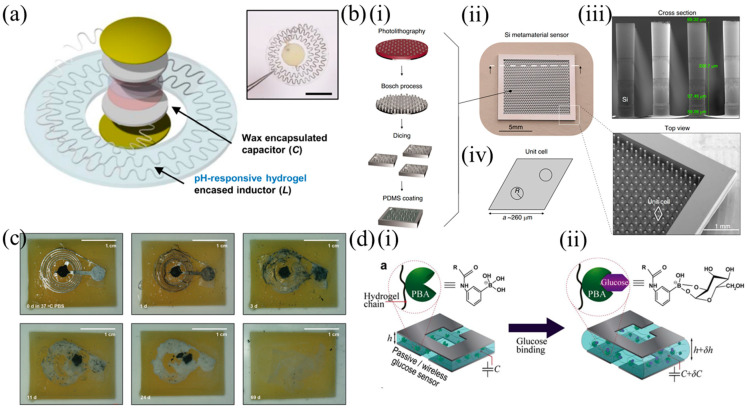
Examples of passive implantable sensors that utilize different material properties for the detection of pH, temperature and glucose through changes in polymer or hydrogel properties. (**a**) The bioresorbable pH sensor for gastric leakage detection of [53]. The device consists of a serpentine spiral inductor, which is encased within a pH-responsive hydrogel. The circuit is completed with a wax-encapsulated capacitor. Copyright © 2024 The Authors. Distributed under the terms and conditions of the Creative Commons Attribution (CC BY 4.0) license (https://creativecommons.org/licenses/by/4.0/). No changes were made. (**b**) An acoustic metamaterial-based temperature sensor [70]. Temperature variations change the bulk modulus of PDMS and Si, producing a shift in the resonance frequency. (**i**) The fabrication process of the device. Steps include deep reactive ion etching (DRIE) of a 4-inch, 500 μm-thick Si wafer and coating of polydimethylsiloxane (PDMS) as a polymeric matrix. (**ii**) The fabricated device. (**iii**) Scanning electron microscopy (SEM) details of the fabricated silicon micropillars. The micropillars had a nominal height of 350 μm and nominal radius of 35 μm. (**iv**) The unit cell and its arrangement. © The Authors 2024. Distributed under the terms and conditions of the Creative Commons Attribution (CC BY 4.0) license (https://creativecommons.org/licenses/by/4.0/). No Changes were made. (**c**) The temperature sensor proposed in [71], consisting of an LC circuit with a temperature-sensitive PEG capacitor. The images show the in vitro biodegradation process of the device in PBS at 37 °C. © 2020 WILEY-VCH Verlag GmbH & Co. KGaA, Weinheim. Reproduced with permission from John Wiley and Sons. (**d**) The passive hydrogel-based glucose sensor demonstrated in [66]. (**i**) Illustration of the structure of the device and the broad-side coupled, split-ring resonator interceded by the p(PBA-co-AAm) hydrogel interlayer. (**ii**) Illustration of the swelling induced to the interlayer in the presence of glucose binding with PBA. Swelling of the interlayer and changes in its thickness, changes the capacitance of the resonator. Upon glucose uptake, the hydrogel swells, increasing the capacitance of the device. Reprinted from Biosensors and Bioelectronics, Vol 151, M. Dautta, M. Alshetaiwi, J. Escobar, P. Tseng, Passive and wireless, implantable glucose sensing with phenylboronic acid hydrogel-interlayer RF resonators, Pages 112004, Copyright (2020), with permission from Elsevier.

A different approach for detecting pH levels was presented in [53] (Table 1) and is shown in Figure 6a. In this case, a transient LC circuit was fabricated to detect gastric leakage during the critical period of seven days following laparoscopic sleeve gastrectomy (LSG). Leaked contents from the stomach are highly acidic (pH ~1 to 3) compared to other abdominal fluids, thus a pH sensor would be able to detect such a situation. The inductive coil was implemented with a serpentine structure of Zn, coated with a thin layer of bioresorbable wax. A pH-responsive hydrogel (poly [2-(diisopropylamino)ethyl methacrylate] and poly(ethylene glycol)diacrylate (PDPAEMA-PEGDA) copolymer), with high selectivity, that was both biodegradable and mechanically robust, encased this inductor. An increase in pH level caused swelling of the hydrogel that changed its volume, which in turn led to the deformation of the inductor. This caused a measurable shift in the sensor’s resonant frequency. The parallel-plate capacitor was implemented with the use of a bioresorbable PLGA film as the dielectric layer, sandwiched between a pair of Zn electrodes. The 3D structure was thermally bonded via a poly(vinyl alcohol) (PVA) adhesive. The capacitor was connected to the inductor with a bioresorbable conductive paste composed of a mixture of candelilla wax and W microparticles. The device was shown to operate at 20 MHz.

A MEMS diaphragm-based rigid RLC pH sensor, where a hydrogel swelling as a function of pH concentration deflects one of the capacitor plates, changing its resonance frequency, was presented in [112]. Prior to hydrogel loading, the sensitivity of the device to applied pressure was equal to 222 kHz/kPa between 41.9 and 51.1 MHz. With the pH-sensitive hydrogel, the sensor sensitivity was 1.16 MHz/pH for pH 3.0–6.5. Nevertheless, the response time of the sensor was rather long and equal to 45 min. The inductor had 20 turns, an external length of 5mm and an inductance of 2.82 μH.

Magnetoelastic sensors were introduced earlier. An interesting alternative is to use such sensors with pH-sensitive polymers and hydrogels that change their mass depending on local pH. In response to an applied magnetic field, the magnetoelastic sensor mechanically vibrates at a characteristic frequency that is inversely related to the mass of the attached polymer layer [113,114,115]. Such approaches have not been demonstrated to our knowledge in implantable applications; however, it is worth noting them to stimulate future developments.

Another approach involves the use of standard solid-state electrochemical potentiometric sensors [116]. This was demonstrated in [117], where an RLC circuit was presented that comprised an inductor in parallel with a voltage-sensitive capacitor (i.e., a varactor) biased by the resulting potential difference between an Ag/AgCl reference electrode and an antimony/antimony oxide pH-sensitive working electrode. With changing pH, the potential across the ion-selective sensor changed, changing the capacitance of the varactor and thus the resonant frequency of the circuit. Screen printing was used to pattern silver ink on a thick alumina silica substrate, to form the inductor, the interconnects forming the circuit and the electrodes. The ink was cured at 800 °C. After temporarily insulating all features with a cellulose film apart from the reference electrode, the device was dipped in sodium hypochlorite solution for 1 min to create an AgCl layer. A KCl layer was then screen-printed on the electrode and the device was again cured at 750 °C. Antimony pieces were placed on the working electrode and the device was then baked at 700 °C to melt the antimony. When cooled, a thin oxide layer was formed. Screen printing was then used to print an insulating layer, on top of which conductive lines were printed to connect the inductor pads to the electrochemical electrodes. The device was further insulated with a spray-coated enamel layer. The pH sensor achieved a sensitivity of 50.43 mV/pH, with a drift of about 4% of the full-scale output in 5 pH cycles, and a response time of 30 s. The sensor was developed for Gastroesophageal reflux disease (GERD), where a malfunctioning lower esophageal sphincter causes gastric liquid to translocate into the esophagus from the stomach. This can cause heartburn, chest pain, dyspepsia, dysphagia, cough and asthma and can lead to esophageal stricture, hemorrhagic esophagitis and esophageal cancer [117].

A wireless passive pH and temperature sensor that allows temperature-compensated remote pH monitoring was presented in [118]. The sensor was quite large and not intended to be implanted; however, its principle of operation is worth noting, as it could be applicable to implantable applications. The sensor consisted of a passive RLC resonator the resonant frequency and quality factor of which were, respectively, pH- and temperature-sensitive. The sensing circuit was an inductor connected in parallel with a temperature-dependent resistor (i.e., a thermistor) and a voltage-dependent capacitor (i.e., a varactor). An Ir/IrOx solid-state pH-sensing electrode and an Ag/AgCl reference electrode connected in parallel with the other circuit elements provided the biasing voltage of the varactor. Thus, changes in pH changed the capacitance and thus the resonant frequency of the resonator, while changes in the temperature changed the resistance of the circuit which affects the Q-factor. In this way, both parameters could be measured through the same simple circuit. The pH sensor demonstrated a Nernstian sensitivity of −58.19 mV/pH at 25 °C. The demonstrated sensor prototype operated between 17 GHz and 20 GHz. Thus, this sensor was able to measure two quantities, pH and temperature, with the latter being the focus of the following section.

### 2.4. Temperature Sensors

Another category of wireless, passive, implantable sensors are temperature sensors. These devices are of significant medical importance as regional internal body temperature is a marker for immune response anomalies and wound healing transient processes. On top of that, these measurements can help monitor the progress of hypo- and hyperthermia treatments. It has also been suggested that temperature is an indirect way of monitoring post-surgery infection. To that end, a permanent implantable temperature sensor, shown in Figure 6b, was proposed in [70] (Table 1). In this work, an array of Si nanopillars embedded in PDMS was developed to form an acoustic metamaterial. The ultrasound reflection of this metamaterial-based device strongly depends on temperature. So, temperature differentials of as low as 30mK were shown to be detectable by using a commercially available ultrasonic transducer at 5 MHz, with a sensitivity of 2.9 × 10^−3^ K^−1^. The device consisted of silicon micropillars arranged in a hexagonal lattice and PDMS, two contrasting acoustic impedance materials. The micropillars were formed with deep reactive ion etching (DRIE) and the overall design was optimized to exhibit an acoustic reflectance resonating at ~5 MHz. Silicon’s acoustic impedance (~ 22 MRayl) is one order of magnitude larger than that of water or soft tissues (~ 1.7 MRayl). This large mismatch allows a high signal intensity. The temperature sensitivity of the device was primarily due to the temperature dependence of the bulk modulus of PDMS. This determines the speed of sound of the longitudinal (p-) waves in PDMS.

A transient approach for detecting temperature over clinically relevant time scales was presented in [71] (Table 1). In this approach, presented in Figure 6c, an RLC circuit was created using bioresorbable materials such as polyethylene glycol (PEG) and Mg in a wax housing. The capacitor of the circuit was composed of the Mg electrodes and the temperature-sensitive PEG dielectric. This material’s dielectric constant depended on the temperature and so temperature changes led to dielectric constant changes which, in turn, led to capacitance changes and resonant frequency changes. The device was shown to operate at 70–90 MHz with a 4-day stability period.

A permanent passive temperature sensor for orthopedic applications was presented in [119]. The device was designed to be embedded within orthopedic implants, such as an interference screw, and to monitor temperature post-surgically as a marker of infection. Interference screws (e.g., sized 8 mm × 12 mm) are typically non-metallic polymeric devices, used to anchor grafts in tendon/ligament tear reconstruction surgeries. These are common sports-related injuries that are pervasive in young athletes. The sensor was once again an RLC circuit, composed of commercial off-the-shelf components (L = 310 nH and C = 100 pF). The sensor had the potential to monitor internal wound temperature for the diagnosis of local infection at the implant site, which can occur even two years after the operation. Infections can lead to implant failure, tissue necrosis, and amputation. Current approaches are slow, tedious, and non-specific, while thermographic imaging is limited to surface or near-surface wounds. The proposed sensor employed a thermistor (22 Ω at 25 °C) as the temperature-sensitive element and could measure temperature reproducibly within 30–42 °C. Since Rt changed relatively linearly with temperature within the range of 30–42 °C, the Q factor was expected to exhibit a linear temperature response.

In [120], the authors proposed an embedded ultrasound system to monitor implant fixation and temperature as an indicator of infection in total knee arthroplasty. The proposed solution requires only a piezoelectric transducer and a 10 mm diameter coil to be implanted. Pulse-echo responses were elicited via a three-coil inductive link, where one of the coils was the implanted one and the other two operate as transmitting (Tx) and receiving coils (Rx). A tone-burst by the Tx circuit excited a pulse-echo response in the implanted circuit via inductive coupling. This was subsequently picked up by the Rx. Knee implants are typically fixed with 2 mm thick bone cement. When this interface is in a good state and an ultrasonic pulse hits it, a fraction of its energy is reflected, and a fraction is transmitted through the interface. If this interface is faulty, it is also infiltrated by joint fluid. Fluids do not sustain shear waves, and thus no pulse energy is transmitted and all is reflected. Thus, implant loosening is detected as a rise in the reflected energy. The ultrasonic shear-wave velocity follows a linear relationship with temperature which is measured by the arrival time of successive echoes.

The coupled elements allowing wireless interrogation can however be substituted with ultrasonic transducers [121]. Pulse-echo measurements are typically employed in such approaches, which is similar to what is often followed in RF-based systems as well. The implanted device is excited at a certain frequency and the oscillation of the resonator at this forced frequency is then converted into a free oscillation at the resonator’s resonant frequency with an exponentially decaying amplitude [121]. This approach was demonstrated in [121] with the use of a temperature-sensitive tuning fork resonator at ~220 kHz, an ultrasonic transducer and a transformer to match the impedance between the two. The echo within a period of 105 ms was analyzed following the pulsed excitation for about 60 ms.

### 2.5. Glucose Sensors

The continuous monitoring of the glucose level is of essential importance to diabetes patients, which are expected to reach 643 million globally by 2030, with three out of four adults with diabetes living in low- and middle-income countries [122]. The most common commercially available methods today consist of sampling the blood by pricking the patient’s finger at almost daily intervals. This method is not only painful and expensive, since it involves consumables, but it is also very much intermittent. As mentioned earlier, semi-implantable glucose sensors, which have to be replaced every couple of weeks, have also become available, as well as fully implantable sensors that also have to be replaced at regular intervals. These provide continuous glucose monitoring and are active devices. Glucose sensing provides a perfect example of the potential advantages of implantable, passive and wireless sensors. In this case, a cost-effective way of having continuous glucose monitoring without consumables or bulky equipment on the patient’s body would greatly improve the quality of life for people suffering from diabetes, particularly in low-income countries. The sensors in this category are required to provide measurements over the patient’s lifetime and so they are designed as permanent sensors. One such fully passive glucose sensor example consisted of metal coils that were integrated on either side of a glucose-sensitive hydrogel. The hydrogel absorbed glucose, changing its volume, thus changing the device capacitance and resonant frequency [66] (Table 1). This flexible sensor (Figure 6c) was composed of Al sheets on Ti sheets that were patterned to form unanchored, capacitively-coupled split-ring resonators on supporting vinyl sheets using an electric knife cutter. A glucose-responsive (PBA-co-Aam, phenylboronic acid hydrogel, with 4% polyacrylamide) hydrogel was then deposited on top and another ring resonator structure was placed on top, making a sandwich. After hydrogel polymerization at room temperature, the vinyl support was removed and the sensor was kept in phosphate-buffered saline (PBS) for one day. The device measured 5 mm × 5 mm × 250 μm. The thinner the hydrogel interlayer, the faster the response time of the sensor. For the examined arrangement, this was 5 min. In addition, hydrogel swelling was pH-sensitive, with optimal sensitivity found to be between pH 7 and 7.5. A lower percent mass exhibited enhanced swelling and thus a more responsive sensor. The resonant frequency of the metallic structures was designed to lie within 400 MHz and 800 MHz and the sensor sensitivity was found to be 304 kHz/(mg/dL). A light-emitting diode (LED) was also added as an alternative readout scheme, where light intensity was correlated to glucose concentration. The small size and low operating frequency of the device was facilitated by the broad-side coupling of the resonator and the high dielectric constant of the hydrogel (close to 80). The sensor was interrogated using a network analyzer and an H-loop antenna and over a 45-day period it exhibited no signal drift or sensor response degradation.

A second sensor designed to measure glucose levels in interstitial fluid (ISF) was presented in [67] (Table 1). Glucose content is much higher in ISF than in the sweat or saliva, making it a more appropriate medium to monitor glucose levels. There is a high correlation between ISF glucose level and blood levels. The ISF dielectric permittivity changes as the concentration of blood glucose changes over a broad frequency spectrum. The proposed sensor monitored this change in dielectric permittivity. This was reflected as a change in resonance frequency of the sensor. This device was shown to operate at 2.2 GHz. This band was chosen by considering the characteristics of the dielectric permittivity of water and tissue across frequency. For example, at the low GHz range, tissue permittivity is relevantly constant. The subcutaneous-implantable electromagnetic resonator sensor was composed of Au lines on a Teflon substrate with a biocompatible polyolefin and polyamide packaging. It was designed as a planar sinusoidal modulated resonator to achieve electromagnetic-induced transparency (EIT)–like transmission in the resonance window. The proposed EIT-based sensor was a non-radiating sensor that had a strong oscillating near-field which enhanced the dielectric sensing performance and that had ultra-high Q resonance. It monitored changes in the dielectric constant of ISF as a result of glucose level differentials. A sensitivity between 41 kHz/mg dL^−1^ and 261 kHz/mg dL^−1^ was demonstrated.

### 2.6. Less Common Sensors

#### 2.6.1. Tissue Conductivity and Bioimpedance

A very different approach to developing passive thread-like implantable sensors that can be deployed through catheterization or injection was presented in [68] (Table 1). The application under consideration in this case was the monitoring of the electrical conductivity of the lungs for detecting congestive heart failure. Electrical bioimpedance is a powerful technique applicable in a wide range of applications, where a physiological phenomenon is encoded in the electrical properties of tissues [123]. The sensor consisted of a thin elongated body composed of a diode in parallel with a resistor, which were in series with a capacitor, with two electrodes at each end. The capacitor also acted as a DC blocking capacitor, protecting the electrodes from damage. Alternating high frequency (>1 MHz) current bursts injected by on-skin electrodes reached the diode through volume conduction. Due to rectification of these bursts at the implant diode, the capacitor was charged. By exciting the implant with signals greater than 100 kHz, stimulation of excitable tissues was avoided. However, frequencies greater than 5 MHz were recommended [68]. The distance between electrodes was set to 25 mm; however, this will need to be increased to the order of a centimeter or more. After the end of each burst, the capacitor discharged through the resistor and the surrounding tissue and the current reached the interrogation unit through volume conduction. This was a decaying voltage signal across the implant electrodes, which could be sensed by the external unit across the pair of external electrodes. The reader also included switching diodes, such that when the excitation burst is off the transient response of the implant, it can be recorded by the external interrogator for further analysis. Analysis of the signal consisted of finding the time constant of the recorded signal, which was in this case a function of the resistance and capacitance of the implant as well as the tissue impedance (if electrode impedance and tissue reactance are ignored then τ = C[R + 1/σπD}, where D is the electrode diameter).

#### 2.6.2. Proteolytic Activity

Proteases are considered a marker for the existence of microbial contamination and infection in the human body. So, monitoring their presence has enormous value for a limited time following a discrete medical procedure. With that in mind, a transient passive sensor for monitoring proteolytic activity was developed in [69] (Table 1). A crosslinked fatty acid-coupled gelatin network with incorporated caprylic acid was created. A film of this composite was coated onto commercially available screen-printed gold electrodes with an LC circuit connected to them. The proteolytic degradation of the gelatin led to a change in the resistance of the device, which was translated into a change in the Q-factor and the LC resonant frequency. This frequency was designed to be around 15 MHz. This particular device followed in concept devices that utilize their material’s transiency as the means of detecting a substance or physiological state.

#### 2.6.3. Neural and Other Electrophysiological Signal Recording

**Figure 7:**
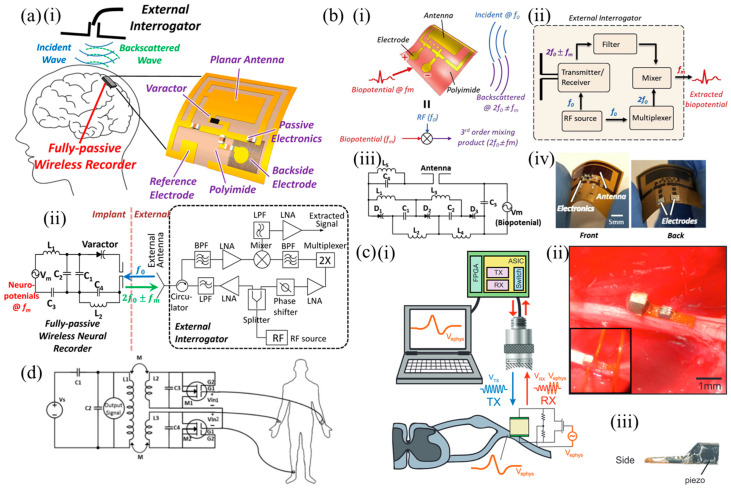
Examples of different approaches for measuring bio-potentials, where backscattering is exploited, as well as varactors or single transistors and ultrasonics for measuring voltage signals. (**a**) (**i**) Illustration of the architecture and the use of a flexible permanent passive device capable of measuring voltages through the use of a varactor. It mixes the radio frequency (RF) carrier signal with the neuropotentials to create third order products. The signal is then backscattered to the external interrogator. Filtering and demodulation allow extraction of the neuropotentials. (**ii**)_Schematic of the implantable device and of the external interrogator [72]. Reprinted with permission from S. Liu et al., “Fully Passive Flexible Wireless Neural Recorder for the Acquisition of Neuropotentials from a Rat Model,” ACS Sens., vol. 4, no. 12, pp. 3175–3185, Dec. 2019, doi: 10.1021/acssensors.9b01491. Copyright 2019 American Chemical Society. (**b**) Another example of a passive device capable of recording electrophysiological signals [124]. (**i**) Architecture and operational principle of the device. (**ii**) Schematic of the architecture of the external interrogator and (**iii**) the implanted device. (**iv**) Images of the fabricated flexible sensor. Reprinted from Biosensors and Bioelectronics, Vol 139, S. Liu, X. Meng, J. Zhang, J. Chae, A wireless fully-passive acquisition of biopotentials, Pages 111336, Copyright (2019), with permission from Elsevier. (**c**) The ultrasonic neural dust approach from [128], where following a pulsed excitation, the backscattered signal is recorded and analyzed to extract the neural signal. (**i**) Architecture of the system. (**ii**) Image of the implanted device. (**iii**) Side image of the device. Reprinted from Neuron, Vol 91, D. Seo, R. M. Neely, K. Shen, U. Singhal, E. Alon, J. M. Rabaey, J. M. Carmena, M. M. Maharbiz, Wireless Recording in the Peripheral Nervous System with Ultrasonic Neural Dust, Pages 529–539, Copyright (2016), with permission from Elsevier. (**d**) The approach proposed in [129] to record differential electrophysiological signals. Similarly to the neural dust approach, transistors are used. Copyright © 2023 The Authors. Distributed under the terms and conditions of the Creative Commons Attribution (CC BY 4.0) license (https://creativecommons.org/licenses/by/4.0/, accessed on 23 December 2024). No changes were made.

A valuable way of understanding brain functionality and diagnosing neurological diseases is by recording neural potentials in the brain. For such an application, it is essential that the sensor exhibits the necessary flexibility, on top of the other common requirements for all implantable and wireless passive sensors. In the work of Liu et al., this requirement stems from the need for attachment on the skull below the scalp [72] (Table 1). The curved morphology of the skull dictates a flexible device for maximal conformity, proper sensor operation and patient comfort. This sensor (see Figure 7a) was designed to monitor neuropotentials at the brain and occupied an area of 9 mm × 8 mm × 0.3 mm. The wireless neural recorder was fabricated on a 90 μm-thick polyimide substrate using a standard flexible PCB process and was permanent and fully flexible in nature. It was composed of an RLC circuit that contained a single-layer planar antenna, a backside electrode, three capacitors, an inductor, and a varactor. One of the capacitors functions as a DC-blocking capacitor, protecting the tissue from the DC component from the varactor. An external circuit transmitted a signal at 2.32 GHz. Changes in varactor potential and, so, capacitance due to neuropotentials on top of the transmitted signal altered the resonant frequency of the device. Different value capacitors and inductors were used to set different resonant frequencies for different sensing nodes and to thus isolate and multiplex neurophysiological recording from different locations. The minimum detectable signal was found to be 60 μV_pp_. Another example of a flexible fully passive device for biopotential recordings was presented in [124]. The device, shown in Figure 7b, was fabricated on a 90 μm-thick polyimide substrate measuring 18 mm × 15 mm × 0.5 mm. Once again, backscattering was exploited. Biopotentials were modulated by an array of varactors with the incoming RF carrier signal (2.33 GHz), that was backscattered to the external reader [124]. Both in [72] and [124], machine learning was employed to aid in the interpretation of the results. In this case, biopotentials as low as 250 μV_pp_ were detectable. In [125], the implant and interrogator had highly efficient dual-band antennas that radiate at 2.4 GHz and 4.8 GHz. The size of this device was 10 mm × 9 mm. The 2.4 GHz carrier signal turns on the two anti-parallel diodes of the implant, where it was harmonically mixed with the neural signal. The output of this mixer was a signal at ~4.8 GHz, which was backscattered to the interrogator for demodulation and analysis. Similar examples were presented in [126,127].

At this point, it is worth mentioning the neural dust approach initially demonstrated by [128,130], although it violates the definition given in this paper for a fully passive sensor, according to which such a device should not have any active components, such as transistors. As shown in Figure 7c, this example utilized a single transistor, two resistors and a piezoelectric ultrasonic transducer, exploiting ultrasonic backscattering to interrogate implanted mm-scale devices in tissues. The resistors were connected together in series and between the source and drain terminal of a MOSFET, which were also connected across the ultrasonic transducer. The biopotential was then applied between the gate of the transistor and the common terminal of the two transistors. Similarly to other examples discussed earlier, the external device switched between excitation and recording modes. It first emitted six 540 ns pulses every 100 ms and it then recorded reflected pulses. The exciting signal caused the implanted transducer to vibrate, converting the mechanical energy into electrical energy, which in turn supplied the transistor. Simultaneously, the biopotential modulated the transistor’s gate, which changed the current between transducer terminals and altered the vibration of the crystal and the intensity of the reflected ultrasonic energy. In this way, electromyogram (EMG) and electroneurogram (ENG) signals were recorded from anesthetized rats. The authors highlighted that the speed of sound is 10^5^ times lower than the speed of light in water. This leads to much smaller wavelengths at similar frequencies, leading to excellent spatial resolution. In addition, the attenuation of ultrasonic signals transmitted through tissue is significantly less than that of EM radiation [128]. This allows greater penetration depths for a given power. The very small size that can be achieved with this approach allows for several such miniature implants to be deployed. Beamforming approaches can then be applied at the interrogator to focus the interrogating signals to specific directions [131].

Although not common, resistive sensors are also used, where change of the resistance of the RLC circuit leads to changes in the Q-factor and a change to the recorded signal amplitude. Heart rate using a pressure sensor, temperature using a thermistor, and pulse oximetry using a light-dependent resistor were measured using this approach using off-the-shelf components. The disadvantage of this method is that the alignment and the distance between the two coils need to be constant to properly interpret the sensor response. In another approach, shown in Figure 7d, RLC circuits with two depletion-mode N-channel dual-gate MOSFETs were used to measure differential signals that change the loading of the two secondary coils according to the ECG signal [129]. The biopotential signal changed the current flowing through the transistors and thus changed the resistance of the RLC circuit. The resonant frequency essentially stayed constant and the resonant peak amplitude changed due to the changing Q-factor. As mentioned before, the disadvantage of this approach is that the implanted and external coils need to be aligned and at a constant separation. This allowed the common-mode signal to be canceled out and to record signals as low as 40 µV. Similar examples were demonstrated in [132].

#### 2.6.4. Implant Micromotion Sensing and Localization

This category does not necessarily involve an implantable sensor, but rather it describes techniques that can be used to monitor the motion and location of passive implanted devices using an external reader. However, these are methods that can be combined with several of the aforementioned sensors as an added functionality. In [133], an eddy current loop coupled with a tunneling magneto-resistor was used as a displacement sensor to monitor orthopedic implant micromotion. It is estimated that about 10% of orthopedic implantable devices for partial or complete joint replacements require re-operation at some point. Aseptic loosening, mechanical failure, periprosthetic fractures and infections are common reasons for re-operation. High relative motion (>150 μm) between implant and bone leads to ingrowth of fibrous tissues rather than bone. To measure with high accuracy the micromotion of the metallic orthopedic implant, the authors proposed the combination of two sensing methods, an eddy current loop as a magnetic field generator and a tunneling magneto resistor (TMR) sensor placed at its center, such that changes in magnetic field could lead to greater changes in resistance. The current passing through inductive loops created a magnetic field that, in turn, produced eddy currents on the surface of a metallic implant placed nearby. The eddy currents produced a secondary magnetic field with an opposite direction to the primary magnetic field. This was translated into a reduced inductance and increased resistance of the loop. Change in the position of the target changed the loop impedance. The TMR sensors consisted of an insulator film sandwiched between the ferromagnetic layers. Depending on the external magnetic field, electrons could tunnel through the insulating layer, forming a resistor. The resistance of TMRs could change by more than 100%. The device operated at 10 MHz. This was not an implantable device; however, it works together with a metallic implant to monitor its location. A similar inductive-based method was demonstrated in [134].

Another interesting method that is worth mentioning here is the use of radar sensing for the localization of markers implanted in the body. This technique can be exploited and combined with several of the aforementioned examples, e.g., for the localization of sensors implanted in various parts of the body forming a multi-sensory network. The Scout^®^ radar sensor of Merit Medical allows surgeons to precisely target tissues of interest, pinpointing its location within 1 mm. In this way, radiology workflow is improved and operating room delays are significantly reduced [135,136]. This reduces costs and clinician workload, and improves patient outcomes, by allowing more successful surgeries. This translates into optimized breast conservation strategies and enhanced outcomes for women. The Scout process can be split into three phases. First is the detection phase, where the radiologist detects areas of interest. This is followed by the implantation of the 4 mm Reflector device concurrently with the biopsy. The Reflector is used to mark soft tissue, including lymph nodes. The device antennas are made of super-elastic nitinol alloy, a commonly used material in medical devices. The Scout reader allows activation of the Reflector and real-time distance measurement with a 60 mm detection range and a ± 1 mm accuracy.

## 3. Flexibility, Stretchability, Transiency and Biocompatibility

Through the previous discussions, several devices were discussed that were flexible, stretchable and transient. The aspects of flexibility, stretchability, transience, packaging and biocompatibility are crucial for implantable devices and have already been reviewed by several other papers. A detailed discussion of these subjects is thus beyond the scope of this paper. However, a general introductory overview is provided here and the interested reader is referred to these other sources for more detailed discussion.

### 3.1. Transiency

The choice of transient bioresorbable materials, devices and their optimization and features have been reviewed in detail over several reviews and seminal papers that discuss different metals, semiconductors (e.g., ultrathin films of silicon and metal oxides) and dielectric materials that can be bioresorbable. Also, techniques for controlled, stimulus-responsive transiency or to define device lifetime by appropriate design are presented [17,19,137,138,139,140,141,142,143]. Bioresorption can be a passive, slow, gradual process that takes place on its own from the moment of implantation, or it can take place with a controlled rate according to appropriate material design (e.g., geometrical design or crystallinity). It can also occur on demand and in a triggered manner where well-defined triggering events initiate the transient response according to some form of stimulus (e.g., controlled exposure to solvents, water, heat, light, or electrical current) [17,143].

Biodegradable, bioresorbable transient materials for medical application must be biocompatible and must degrade into products that can be metabolized or removed from the body via natural pathways [56]. The rates for complete disappearance of such devices and materials depend on temperature, pH, ionic content, material thickness, the deposition method used and the material’s resulting stoichiometry and morphology. They can range from minutes and hours to weeks and months.

The most well-known biocompatible and biodegradable metals are Mg and Fe. Other options include Zn, W and Mo. They dissolve via hydrolysis and reported dissolution rates include 1700 nm/day for Mg, ~3500 nm/day for Zn, ~150 nm/day for W, ~80 nm/day for Fe and ~20 nm/day for Mo [140]. Metallic alloys also dissolve via hydrolysis. For example, MgZn alloy (AZ31B) achieves a rate of 480 nm/day. The transiency of these metals has been extensively investigated in [141].

In terms of semiconductors, it has been found that monocrystalline silicon naturally dissolves in biofluids. Silicon nanomembranes (NM), when immersed in PBS (pH 7.4) at physiological temperature (37 °C), dissolve at a rate of ~5 nm/day [140]. This relationship between dissolution and thickness is linear. Typically, Si NM for the discussed applications are about 100 nm thick, which would mean that they would completely dissolve within approximately 20 days under these conditions [140]. The reaction rate increases with increasing pH, which can be exploited as a means of sensing, as was discussed earlier. Different semiconducting materials dissolve with different rates. Polycrystalline Si, amorphous Si, Ge, and Si-Ge dissolve at rates of 2.8, 4.1, 3.1, and 0.1 nm/day, respectively [140]. ZnO is another inorganic semiconductor that dissolves by hydrolysis. It is a piezoelectric material suitable for mechanical sensing and energy harvesting. It dissolves at a rate of ~4–12 nm/h depending on the deposition process used and thus the crystal structure. A popular semiconductor for flexible electronics is amorphous indium-gallium-zinc oxide (a-IGZO), which also dissolves by hydrolysis. MXenes is a promising novel family of 2D materials that includes transition metal carbides, and either nitrides or carbonitrides. Biocompatibility has been demonstrated for some of them, with few being biodegradable [144].

Dielectric materials are also critical, as we have seen in our previous discussion. Typical materials in microelectronics, such as SiO_2_ and Si_3_N_4_, are also dissolvable by hydrolysis. Their dissolution rate is typically much lower than Si and, as with Si, depends on the deposition method used (and thus stoichiometry), as well as pH, temperature and other factors. Rates of SiO_2_ dissolution of 0.003 nm/day (thermally grown) to 10 nm/day (electron-beam evaporation) have been reported under the same conditions [140]. Silicon nitrides dissolve in two steps: first into SiO_2_, which then further dissolves via hydrolysis as already mentioned. Dissolution rates of 0.16 nm/day (low-pressure chemical vapor deposition, LPCVD) to 0.85 nm/day (plasma-enhanced chemical vapor deposition, PECVD) have been reported. The byproduct of Si-based material dissolution is silicic acid (Si(OH)_4_), which is a nontoxic molecule. It is the most common form of silicon in the human body, with serum levels in the order of 11–25 μg silicon/dL. It does not accumulate in the body and is excreted via the urinary pathway [140].

Several intricate transient devices composed of the materials described above have been implanted in animal models [145]. Histopathological and hematological analyses have shown elevated concentration of the materials in blood and in different organs after a couple of weeks of implantation, as expected, with the levels returning to normal in the following weeks. No “significant inflammation, ischemia/tissue necrosis, or other architectural/histologic abnormalities in the major organs” were detected [140].

In summary, insulating materials can thus be naturally derived or synthetic materials. As highlighted in [146], naturally derived materials include silk, cellulose, sodium carboxymethylcellulose (Na-CMC), alginate, starch, chitosan, albumin, gelatin, keratin, shellac, candelilla wax, galactomannan, carrageenan, agar, polyhydroxybutyrate (PHB), polyhydroxyvalerate (PHV), and poly(3-hydroxybutyrateco-3-hydroxyvalerate) (PHBV). Synthetic materials include PVA, PLGA, PLA, PCL, polyethylene glycol (PEG), polyethylene glycol diacrylate (PEGDA), PGA, polyanhydride (PA), polydopamine (PDA), polybutylene adipate terephthalate (PBAT), poly(trimethylene carbonate) (PTMC), poly(desaminotyrosyl-tyrosine ethyl ester carbonate) (PDTEC), poly(glycerol sebacate) (PGS), and poly(octamethylene maleate (anhydride) citrate) (POMaC) [146]. Bioresorption follows four main mechanisms: (a) dissolution through the interaction of solvated polymer with water, (b) enzyme-catalyzed depolymerization (enzymatic reaction), (c) water-induced depolymerization (hydrolysis), and (d) reactive oxygen (or nitrogen) species (ROS)-induced depolymerization (oxidative reaction) [146]. Typically, dissolution will be combined with one of the other mechanisms. Natural polymers usually degrade through enzymatic reactions, which cleave the polymer chains as biological catalysts. The byproducts of this process typically diffuse in the body through bioaccumulation and mineralization. Aliphatic polyesters chemically degrade by hydrolytic cleavage of backbone ester bonds and enzymatic promotion [56]. Hydrolytic degradation generates oligomers or monomers that can be absorbed in the body [146]. Examples include PLA, and its stereochemical forms poly(L-lactide) PLLA, poly (D-lactide) (PDLA) and poly(DLlactide) (PDLLA); poly(glycolic acid) (PGA); polycaprolactone (PCL) and poly(3-hydroxybutyrate) (PHB), as well as poly (lactic-co-glycolic acid) (PLGA) [56,142]. The properties of PLGA can be tuned by adjusting its molecular weight and the ratio of lactide to glycolide. It undergoes degradation via hydrolysis of its ester linkages. Thus, for example, increasing its lactide content increases the material’s hydrophobicity and decreases water absorption, which in turn reduces its degradation rate. PCL, a widely available material, is hydrophobic and semi-crystalline. The degree of crystallinity can be tuned by molecular weight and by appropriate material processing. Through hydrolysis, the major degradation products of PLA, PGA, PCL, PA and PLGA are small molecules of lactic acid, glycolic acid, and 6-hydroxyhexanoic acid. These are eventually eliminated from the body as carbon dioxide and water [146]. Rice paper is another option due to its low cost, wide availability and suitability as an ingestible material. Rice paper has been employed as a substrate in some examples [147]. Metal nanoparticles can be combined with biodegradable polymers to create biodegradable composites, e.g., PVP with Zn, PCL with Fe and PLA with Mg. The biodegradability of materials employed in transient biomedical devices was recently reviewed in [148].

The work reported in [147] is particularly interesting to highlight, as it is not a sensing device, but a device for thermal therapy. It is important, as it is a completely passive device that can be combined with sensors to create multifunctional systems. For example, one device can detect infection, and accordingly a thermal element can aid in eliminating the source of infection. The device reported in [147] was composed of Mg coils, Si serpentine NM resistive microheaters and an MgO interlayer dielectric on a silk substrate. The device was implanted under the skin, allowing localized temperature increase of up to 20 °C for 10 min when the primary coil was operated at 500 mW and 80 MHz. This increased skin temperature up to 49 °C, leading to significant decrease of bacteria population after 24 h, when implanted at an infected site. The device dissolved completely after 15 days [147].

Similarly, neural tissues can be stimulated with devices such as the one presented in [149]. The device included a bilayer, dual-coil loop antenna (Mg, ~50 μm thick) with PLGA dielectric interlayer, a diode based on a 320 nm-thick doped silicon NM with 300 nm-thick Mg electrodes, and a 50 μm-thick Mg parallel plate capacitor with a 600 nm-thick SiO_2_ dielectric. The nerve cuff electrode could deliver cathodic, monophasic electrical impulses (duration: 200 μs; threshold voltage: 100–300 mV). A similar transient stretchable device was presented in [150]. Thus, fully passive biopotential recording can be combined with passive electrical stimulation to yield a closed-loop system through the external interrogator.

The fabrication of such devices is a complex process, since standard manufacturing approaches (e.g., photolithography and etching) may not be suitable due to the sensitive nature of the materials. In addition, the use of shadow masks limits patterning resolution. Thus, transfer printing/stamping approaches have been deployed, where features manufactured in a temporary substrate that can support standard high-resolution manufacturing techniques are then moved to the final material.

### 3.2. Flexibility and Stretchability

Depending on the location of the implantation site, stretchability might be important. Soft stretchable materials are a crucial family of materials for biomedical devices. Silicone-based elastomers have dominated the field, with advanced formulations increasingly being proposed to address challenges related to stretchability, hermeticity, degradability and biocompatibility. In [151], ultra-stretchable, biodegradable elastomers capable of stretching up to ~1600% were presented. The poly(l-lactide-co-ε-caprolactone) (PLCL)-based elastomers can be tuned by varying the monomer-to-initiator ratios to yield different molecular weights of PLCL elastomers that have different chemical/physical/mechanical properties according to application requirements. The authors demonstrated materials with outstanding toughness and tear-tolerance, and the use of these as a matrix to implement conductive composite elastomers with poly(3,4-ethylenedioxythiophene): poly(styrenesulfonate) (PEDOT:PSS) as the biocompatible conductive filler and N-methyl-N-butylpyrrolidinium as the biocompatible biodegradable plasticizer. The strain-tolerant composites retain high levels of conductivity even under tensile strains of ~550% from an initial conductivity (~220 S/cm) and a robust cyclic stability under 40% strain. By changing the material composition, highly strain-sensitive composites were created. Mo was used as the conductive filler, leading to strain sensors with gauge factors of 5500–7500 within a range of 13% strain, negligible hysteresis and low detection limit (~0.01%). These characteristics were exploited to create epicardial implantable devices for cardiac monitoring. Liquid metals are becoming a popular choice for flexible and stretchable devices (eGaIn and GaInSn) [152,153]. Such materials have been used to form interconnects, complete circuits and sensors, exploiting wetting of materials, the oxide formed on their surface or the use of microfluidic channels for patterning [154]. Coils, antennas and other devices as well as electrodes and sensors that are biocompatible have been demonstrated in the literature for implantable and wearable applications using flexible polymer, stretchable elastomers and hydrogels. Permeable electrospun substrates, as well as composite materials with magnetic microparticles and liquid allowing remote magnetic control and controllable mechanical properties (e.g., stiffness), have also been reported [155,156,157,158,159,160].

Hydrogels are increasingly being used in implantable applications, as they display unique biomimetic properties to tissues. This is because their mechanical properties, high water content, and porosity resemble that of the extracellular matrix. They can be tuned to match specific tissues, promoting a lower foreign body reaction (FBR). The mechanical and chemical mismatch between implanted materials and tissues is a prime trigger for the formation and evolution of the FBR [161]. The most common hydrogels found in the literature include collagen, alginate, gelatin, fibrin, hyaluronic acid (HA), PEG, polyacrylamide (PAAm), poly(vinyl alcohol) (PVA), and poly(hydroxyethyl methacrylate) (PHEMA). Biodegradable hydrogels susceptible to hydrolytic or enzymatic cleavage can also be engineered by incorporation of labile chemical bonds [161]. Such materials can be used as a substrate or as a matrix for the creation of conductive composites [161,162]. Conductive polymers (e.g., polythiophene (PT), polypyrrole (PPy), PANI, poly(p-phenylene) (PPP), PEDOT) or percolating conducting fillers (e.g., nanoparticles, flakes, nanotubes, nanowires) and in general inorganic ions (e.g., acids, bases and metal ions) can be used to create such conductive hydrogels, achieving typical conductivities in the order of 10^–4^–10^2^ S/m [161,163]. In addition, self-healing capabilities can also be introduced by designing reversible weak interactions in the polymer networks. They can also be made physically or chemically responsive when exposed to stimuli (e.g., pH, temperature, light, humidity, pressure or chemical and biological compounds).

The vast majority of stretchable sensors in the literature, however, are not biodegradable. There are a plethora of review papers on the subject of permanent (non-biodegradable) flexible and stretchable sensors for biomedical applications [50,89,164,165,166]. Common substrate materials for flexible embodiments include PET, and especially polyimide, due to its high temperature tolerance and ease of processing. Flexible printed circuits (FPCs) are widely available commercially; however, they primarily consist of copper on polyimide to form interconnects and also sensors, typically with a resolution of 100 μm or greater [167,168,169,170,171]. Commercial processes for stretchable printed circuits (SPC) based on thermoplastic polyurethane (TPU) substrates have also emerged [111]. However, thin film microfabrication technologies and additive manufacturing approaches can be exploited for manufacturing to achieve smaller features, more complex and advanced sensors and also active devices. Indium gallium zinc oxide (IGZO) and organic semiconducting materials (e.g., Dinaphtho[2,3-b:2′,3′-f]thieno[3,2-b]thiophene, DNTT) have been exploited to create flexible active devices with good performance that have also been transitioned into commercialization (see, e.g., Intrinsic Semiconductors, UK) [172,173,174]. Additive manufacturing techniques can nowadays achieve resolutions similar to FPCs and can also be exploited to create active devices and sensing elements [175,176,177,178,179,180,181,182]. Methods such as inkjet, stencil, screen and extrusion-based 3D printing, spray/aerosol coating and transfer printing, as well as gravure, roll-to-roll and other stamping approaches can be exploited using novel micro- or nano-particle conducting inks or silver, copper or gold. Electrochemical and electroless depositions can also be exploited to deposit additional materials in printed structures. These techniques can also be combined together to achieve devices with different features and scales and to process materials that may not be compliant with a process. For stretchability, both conductors and flexible substrates have to be patterned accordingly to introduce stretchability by their geometrical design; for example, with serpentine or horseshoe spring-like structures or even kirigami-inspired architectures [183,184,185]. Interconnects and antennas can be patterned with complex self-similar fractal geometric patterns to withstand elongation in one or more directions [186]. Alternatively, pre-stretched elastomeric substrates can be used with selective bonding of conductive, semiconducting and dielectric layers, allowing, for example, buckling in 3D [187,188]. Another approach involves the use of the island–bridge approach, where rigid or strain sensitive devices sit on islands that are interconnected with stretchable interconnects [166,187]. The mechanical design of such approaches must be performed with careful consideration of the final application and must be combined with computational simulations to understand and optimize the device under elongation and stress. Finally, conductive polymers and conductive composite elastomeric materials can be used to create interconnects or sensing elements that are inherently stretchable and/or flexible [189]. The most common elastomer employed in such devices is PDMS (Sylgard, Dow, Midland, MI, USA), Elastosil (Wacker Chemie, Stuttgart, Germany) and Ecoflex (Poly(butylene adipate-co-terephthalate), Smooth On, Macungie, PA, USA), which come in versions with different mechanical and other properties [190]. PEDOT:PSS is a commonly used choice for conducting polymer, while conductive composites can be made with the use of elastomers combined with conductive micro/nano particles of silver, carbon and other materials [191,192,193,194,195,196,197]. The substrate provides a supporting material for the manufacturing of the various devices and sensors and constitutes a fundamental part of a device. Its choice is critical and must be application-specific. Since it is the foundation of the device, it has typically the largest area and thickness and it constitutes most of the device weight. Many of the aforementioned polymers can be used as substrates, with the most popular choices for flexible devices being polyimide, TPU, parylene-C, liquid crystal polymers and PDMS.

### 3.3. Biocompatibility

Biocompatibility is a complex subject that can be addressed using several methods. The description of the host immune response immediately following the implantation of a foreign body, which is a complex cascade of events, has been reviewed extensively in the relevant literature [15,16,20,21]. The FBR can impede partially or even completely the operation of a sensor (e.g., chemical or electrophysiological). Regardless of the location of the implant, the FBR process results in the complete isolation of the device through the formation of a capsule either due to thrombi or scar tissue. Biocompatibility is thus related with the ability of a material or device to be recognized as a foreign body. In addition, toxicity and carcinogenicity of the device materials and their degradation products must be examined. The simplest method, if the implantable device is to be used only for a short period of time, is to ensure that within that time frame the foreign body response is not too intense, through the appropriate choice of materials or through the administration of pharmaceutical agents. Another strategy involves the use of nano-patterning or chemical modification of the implant surface, aiming at preventing the adhesion of proteins, which is often the first step of the FBR process. A common approach involves the gradual and controlled release of pharmaceutical agents that hinders or slows down the host immune response. This can be done through the gradual release of agents loaded into a host material, or in a controlled on-demand or preprogrammed manner, as already discussed for transient materials. Hydrophilic polymers, such as poly(ethylene glycol) (PEG), poly(ethylene oxide) (PEO) and polyamides, are resistant to protein adsorption. Zwitterionic polymers, e.g., polymers prepared from monomer units of carboxybetaine, sulfobetaine, and phosphobetaine (e.g., poly(carboxy betaine)methacrylate, pCBMA or poly(hydroxyl-2-ethyl)methacrylate, pHEMA hydrogels), adsorb only trace quantities of protein and inhibit thrombus formation when used in vessels [16]. Heparin is an anticoagulant that thus minimizes thrombus formation. Immobilization of heparin on surfaces via non-covalent immobilization limits the need for systemic administration, as heparin gradually leaches from the material to the surrounding blood. However, eventually the available heparin will be exhausted [16]. Alternatively, covalent heparin immobilization provides non-depleting, surface-localized anticoagulation. Unfortunately, this is associated with a low attachment efficiency (<10%), while it also leads to conformational changes in its structure, limiting its performance. Heparin is also associated with a short in vivo half-life and great variability. Heparin-mimicking polymers are an alternative. Another alternative is nitric oxide (NO) release, through the use of NO donors (e.g., N-diazeniumdiolates or S-nitrosothiols) as dopant molecules within polymeric layers [15,16,21]. Donor leaching, related with the formation of toxic species, is a concern, with a more lipophilic NO donor (N-diazeniumdiolate-modified DBHD) having been proposed to limit NO donor leaching. However, NO will eventually be exhausted. One solution is to exploit naturally circulating blood S-nitrosothiols and their catalytic Cu^2+^-mediated degradation to generate NO via Cu(II)-cyclen complexes immobilized within the polymer surface of the sensor. NO has also antimicrobial, angiogenic and anti-inflammatory action. The release of tyrosine kinase inhibitors (e.g., masitinib) is another approach. These are enzymes that induce an anti-inflammatory response. Dexamethasone (DX) is a synthetic anti-inflammatory glucocorticoid hormone. DX-loaded PLGA microspheres immobilized in PVA hydrogels have been proposed [16,20]. As a disadvantage, DX is related with DX-induced ischemia as it inhibits angiogenesis. The co-release of angiogenic stimulators has been proposed to counter this effect. Increasing the micro/nano structuring of sensor surfaces, by increasing the porosity, for example, has also been shown to mitigate DX-related issues. Porosity is well known to reduce the FBR, with pore size in the order of 10–35 μm leading to minimal collagen encapsulation and angiogenesis, while pores in the order of 160 μm invoke a more classical FBR response. Electrospun fibers have also been exploited to mitigate FBR [16]. Structural biocompatibility is related with the mechanical interaction between device and tissue, and it is affected by sharp edges and corners, micromotions and mismatched Young’s moduli [198]. The latter can be addressed by several of the aforementioned soft materials with tissue-like properties, such as hydrogels.

### 3.4. Packaging

Hermetic and non-hermetic packaging of systems is also another vital aspect for permanent implants [198]. As mentioned in [199], “humidity-induced corrosion is one of the most dominant factors in implant electronics failure”. Humidity that condenses to water leads to galvanic corrosion, dissolves inorganic passivation, and leads to swelling-induced failures of polymers, among other issues. It can thus short adjacent tracks that ideally are insulated by water-based swelling of the polymer insulation [199]. Typically, for implantable devices with active electronics, a maximum limit of 5000 ppm water vapor inside the package is allowed [24]. This corresponds to a relative humidity of around 8.1%RH at 37 °C [24]. Many different approaches and materials can be used. Silicone-based elastomers, such as PDMS, and parylene-C are the most common materials used for packaging of permanent implantable devices [198,199]. Sensors can also be co-integrated to assess hermeticity, such as capacitive interdigital sensors coated with insulating polymeric (e.g., polyimide) or other materials (desiccant-based composites) that will swell or their dielectric constant will change due to water adsorption, thus leading to measurable changes of capacitance [24,200]. Alternatively, the resistance of metal tracks could also be used as a means of assessing humidity. Such sensors can be implemented in a fully passive approach. The use of ceramic materials in combination with thin film technology and multi-layer implementation represents an area of major interest with significant space for innovation [201]. Sterilization is a final important step prior to implantation and has to be done in such a way that the device is not damaged during the process, e.g., using radiation, or gas sterilization using ethylene oxide [64,198].

The long-term clinical performance of passive implanted devices is something that is generally missing from the relevant literature. The reason for this is that most examples are proof-of-concept academic developments that demonstrate in vitro measurements. Nevertheless, the biocompatibility and biodegradation of the various materials used in such devices using animal models have been well-documented in the literature and were reported in previous sections of this paper [202]. Implantation studies showcasing the efficacy of various devices in performing as intended without inducing inflammatory or other negative effects range from three to four days [60,64,71] to three to 4.5 months [58,86] for fully passive devices discussed in the paper.

## 4. Readout of RLC Resonators

Most papers in the literature focus on the design, fabrication and experimental validation of fully passive RLC-based implanted sensors. The experimental validation is thus achieved primarily using expensive and bulky benchtop instruments such as impedance and network analyzers connected to antennas/coils. Nevertheless, this is not a realistic scenario for real-life applications. There are some examples in the literature where custom hardware is demonstrated in an effort to reduce the overall size and power consumption, or to introduce new capabilities and functionalities. One such example was presented in [203]. The insertion of additional LC tanks within the coupling path of a pair of LC circuits has also been investigated and has been shown to improve sensing performance [204]. A figure of merit (FoM) has been introduced to aid in the development of passive wireless links aimed at maximizing SNR and sensitivity at a desired reading range. This FoM allows the design of optimal geometries of the readout (*L_r_*) and sensing (*L_s_*) coils, the operating frequency (*f_0_*) and the sensor capacitance (*C_s_*). The interested reader is referred to [205] for a detailed discussion of this FoM and its mathematical derivation. Experimental and simulated studies indicated that the proposed FoM improved sensitivity. The capacitance of a typical parallel plate capacitor is defined by

(1) $$C_{p}=\frac{\varepsilon_{r}A}{d}.$$

The capacitance of an interdigital electrode (IDE) planar capacitor is defined as the sum of the IDE capacitance (*C_IDE_*), the substrate capacitance (*C_sub_*) and the sensing capacitance (*C_sens_*):

(2) $$C_{s}=n\cdot l\cdot \left(C_{IDE}+C_{sub}+C_{sens}\right),$$

where

(3) $$C_{IDE}=4\varepsilon_{o}\cdot \frac{K\left(k_{1}^{\prime }\right)}{K\left(k_{1}\right)},$$

(4) $$C_{sub}=2\varepsilon_{o}\cdot \left(\varepsilon_{sub}-1\right)\cdot \frac{K\left(k_{2}^{\prime }\right)}{K\left(k_{2}\right)},$$

and

(5) $$C_{sens}=2\varepsilon_{o}\cdot \left(\varepsilon_{mat}-1\right)\cdot \frac{K\left(k_{3}^{\prime }\right)}{K\left(k_{3}\right)},$$

where n is the number of IDEs, l is the overlapping length, ε_0_, ε_sub_, and ε_mat_ are the permittivities of air, substrate, and applied material, respectively, while *K*(*k′_1_*)/*K*(*k_1_*), *K*(*k′_2_*)/*K*(*k_2_*), and *K*(*k′_3_*)/*K*(*k_3_*) are the ratios of complete elliptic integrals for air, substrate, and sensing film, respectively, based on the geometric parameters of the IDE [205]. If *μ_r_* is the relative permeability, *μ_0_* is vacuum permeability, *d_avg_* is the average diameter, *N_r,s_* is the number of turns, *φ* is the fill-factor, and if *c_1_*, *c_2_*, *c_3_*, and *c_4_* are empirically defined coefficients that dependent upon coil geometry, then the inductance *L_r,s_* of a planar spiral coil (PSC) is defined by the following:

(6) $$L_{r,s}=\frac{\mu_{r}\mu_{o}N_{r,s}^{2}d_{avg}c_{1}}{2}\cdot \left(ln\left(\frac{c_{2}}{\phi }\right)+c_{3}\phi +c_{4}\phi^{2}\right).$$

For circular-shaped spiral coils, *c_1_*, *c_2_*, *c_3_*, and *c_4_* are 1, 2.46, 0, and 0.2, respectively. Spiral inductors are often designed with larger line width (*w*) and smaller line spacing (*s*), to achieve an improved interwinding magnetic coupling and a reduced layout space. For *s* ≤ 3*w*, the maximum error of this analytical equation is estimated to be 8% [205]. If we define *R_DC_* as the DC parasitic resistance of the coil, *t_r,s_* as the metal trace thickness and *δ* as the skin depth, then the parasitic resistance of PSCs is equal to

(7) $$R_{r,s}=R_{DC}\cdot \frac{t_{r,s}}{\delta \left(1-e^{-t_{r,s}/\delta }\right)},$$

where *R_DC_* and *δ* are defined by

(8) $$R_{DC}=\frac{\rho l_{r,s}}{w_{r,s}t_{r,s}}$$

and

(9) $$\delta =\sqrt{\frac{\rho }{\pi \mu_{r}\mu_{o}f_{0}}},$$

where *ρ* is the conductor resistivity and *f_0_* is the operating frequency. The subscripts *r* and *s* stand for readout and sensor. The resonant frequency, *f_0_*, and *Q*-factor for both LC and piezoelectric sensors are defined by the following:

(10) $$f_{o}=\frac{1}{2\pi \sqrt{LC}}.$$

and

(11) $$Q=\frac{1}{R}\sqrt{\frac{L}{C}}.$$

The magnetic coupling between two coils (*L_1_* and *L_2_*) is accounted for by the mutual inductance *M*. *M* depends on the geometry of the two coils and their spacing, alignment and orientation. The magnetic coupling can be described through the coupling factor, *k*, which is a non-dimensional parameter defined as follows:

(12) $$k=\frac{M}{\sqrt{L_{1}L_{2}}}.$$

The response of a passive sensor can be extracted by measuring the phase-dip in impedance spectra, or by measuring the input return loss (*S_11_*) or the real part of the impedance [205,206]. If the impedance of the external inductor is measured, this is equal to the impedance of the external circuit (*L_1_* and the resistance of the circuit *R_1_*) and the impedance *Z_s_* of the couple impedance of the sensor, which consists of inductor *L_2_*, capacitor *C_2_* and the resistance of that circuit *R_2_*, which could be a parasitic resistive element or resistive sensing element [207].

(13) $$Z_{r}=R_{r}+j\omega L_{r}+Z_{s}=R_{r}+j\omega L_{r}+\omega^{2}k^{2}L_{r}L_{s}\frac{1}{R_{s}+j\omega L_{s}+\frac{1}{j\omega C_{s}}}.$$

The parameters of interest *f_0_* and *Q* can be obtained from *Re*(*Z_r_*):

(14) $$Re\left(Z_{r}\right)=R_{r}+\omega^{2}k^{2}L_{r}L_{s}\frac{R_{s}}{R_{s}^{2}+{\left(\omega L_{s}-\frac{1}{\omega C_{s}}\right)}^{2}}.$$

**Figure 8:**
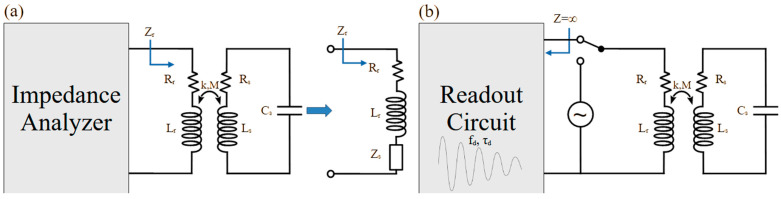
(**a**) Measurement of the impedance from the external primary coil. (**b**) Measurement using a pulsed transient approach. Adapted from [207].

*Re*(*Z_r_*) has a maximum at *f_m_*. This can be found by equating to zero the derivative with respect to *ω* of *Re*(*Z_r_*) and is independent of *k* [207].

(15) $$f_{m}=\frac{2Q}{\sqrt{4Q^{2}-2}}f_{0}$$

with

(16) $$Q\approx \frac{f_{0}}{\Delta f_{m}},$$

where *Δf_m_* is the full width at half maximum of *Re*(*Z_r_*) around *f_m_* [207]. If *Q* is large (>50) then *f_m_* = *f_0_*. In this way, *k* only affects the amplitude. This is illustrated in Figure 8a.

As mentioned before, the passive sensor can also be interrogated in a pulsed transient approach, as illustrated in Figure 8b. This approach has two phases, the excitation and the measurement phase, and a switch as the external coil time divides the coil between the two phases. The input voltage is a damped sinusoid with frequency *f_d_* and a decay time *τ_d_* from which the resonant frequency *f_0_* and the quality factor *Q* can be derived. If *Q* is large, then *f_d_* ≈ *f_0_*, and k only affects the amplitude and not *τ_d_* and *f_d_*, allowing measurement of *f_0_* and *Q* independently of *k* under the defined conditions [207]. If the voltage at time *t* = 0 across the sensing capacitor is defined as *V_c_*, then

(17) $$v\left(t\right)=k\sqrt{\frac{L_{r}}{L_{s}}}\sqrt{\frac{4Q^{2}}{4Q^{2}-1}}\cdot V_{C}\cdot e^{-\frac{t}{\tau_{d}}}cos\left[2\pi f_{d}t-atan\left(\frac{1}{2\pi f_{d}\tau_{d}}\right)\right],$$

with

(18) $$f_{d}=f_{0}\sqrt{1-\frac{1}{4Q^{2}}}$$

and

(19) $$\tau_{d}=\frac{Q}{\pi f_{0}}$$

Parasitic capacitances will affect the measurement accuracy of these methods. In [207], methods for the compensation of the effect of parasitic capacitances on the measurement accuracy were presented.

A method for coupling-independent and robust sensing of fully passive resistive and capacitive sensors that obviates sweeping, permitting single-point sensing and consequently simplifying the design and implementation of the readout system, was proposed in [208,209,210]. The proposed reader implementation exploits a self-oscillating approach and a fast-settling nonlinearity which leads to a voltage amplitude that is proportional to the sensor’s resistance [208,209].

In far-field backscattering measurement approaches, where the reflected wave of an incident excitation signal is measured, one interrogation approach involves the measurement of the third-order intermodulation component. This arises from a non-linear circuit element, which performs mixing of two incident signals of different frequencies, providing their sum and difference frequencies. This was demonstrated in [211]. The reader transmits two frequencies, the sum of which is large enough to be easily filtered, while their difference can stimulate the resonance of a resonant sensor element. The sum is filtered out, while the difference is circulated to a mixing diode, mixing it with the incident signal. This results in several frequency components being backscattered to the reader, with the strongest reflection taking place at the third order intermodulation component. To determine the resonant frequency, the difference in transmission frequencies of the maximal backscattered signal is used. A resonant element of known resonance frequency will be disturbed by a sensing load capacitance, as its capacitance is changed. This allows extraction of the sensor value [211]. Measurements up to 15 m in air were demonstrated.

## 5. Conclusions, Perspectives and Outlook

Passive implantable sensing devices represent a class of devices with great potential. Their simplicity, in that they do not require implanted complex active electronics, is a strong attribute that simplifies the structure of the implant and the related failure mechanisms related with packaging hermeticity, power delivery and cost. The vast majority of devices are pressure- and strain-sensing devices, as they are the simplest quantities that can be interrogated with such passive architectures. They rely primarily on the mechanical deformation of a passive element, typically a diaphragm that acts as one of the plates of a capacitor. However, as we saw, there is a growing body of literature exploiting additional material-based and other functionalities to detect other phenomena. For example, the transiency of a material or the swelling of a polymer/hydrogel, which can be a function of, e.g., pH or temperature. In addition, parameters such as protein and metabolite concentrations can also now be measured, as well as electrophysiological signals. A common characteristic of the discussed examples is that they are capable of interrogating a single parameter. This represents a potential path of future research and novelty. Demonstrating a multiparametric sensing system that is capable of measuring two or more parameters in a passive manner is, to the authors’ knowledge, missing from the literature at the moment. Of course, this can be easily implemented if different sensing devices are implanted far from each other, such that they do not interfere with each other. However, a novel and interesting implementation would aim at a single implantation site, with sensors located on the same substrate, each part of a separate passive circuit, having a distinct resonant frequency around which sensing can be achieved in an approach similar to frequency division multiple access (FDMA), as proposed in [212]. One example leaning towards this direction has been mentioned, where a single RLC circuit was able to compensate for the temperature-sensitivity of pH measurement through the measurement of temperature [118]. Some limited examples where this approach has been exploited to some extent to allow distributed sensing of the same parameter have also been discussed [64,100]. In terms of applications, orthopedic applications seem to be the ones exploiting passive sensors the most. Glucose sensing for diabetics and SSI-related application using transient devices with healing capabilities is another promising research route that can have substantial impact on surgical outcomes. The use of varactors for voltage sensing is a very interesting and promising approach. So far, this seems to have been implemented with off-the-shelf commercially available components. The development of custom microfabricated varactors based on thin-film technology is another path that will aid towards the miniaturization, flexibility, stretchability and transiency of varactor-based approaches. The co-integration of capacitive humidity-sensing capabilities for long-term implants as well as the controlled release of anti-biofouling agents represents important additional functionalities that do not seem to be commonly addressed in passive devices in the literature. These are just some examples showcasing potential paths for further innovation and novelty in the field of passive implantable devices. As was mentioned before, the field of fully passive implantable sensors is lagging with respect to the field of active implants, providing significant opportunities as regards novelty and advanced solutions. This is also true for fully passive ultrasonic devices, with very few examples available in the literature as opposed to devices exploiting a standard inductively coupled RLC circuit.

Manufacturing at scale is essential for any device to be commercially successful and is directly related to the reproducibility of the sensor and the manufacturing process. Clean room-based microfabrication techniques, which are extensively deployed in the microelectronics industry, allow bulk fabrication of high-quality and reproducible devices that closely match each other. The microelectronics design paradigm should be followed where possible in sensor manufacturing. Precise device physics modelling, the inclusion of process tolerances and statistical modelling in the design process and the development of the corresponding design and simulation tools (from schematic design, Monte Carlo and process corner simulations, layout design, electromagnetic modelling and post-layout extracted simulations) are essential to enable high-throughput manufacturing at scale. Well-controlled and primarily automated manufacturing using microelectronics processes is well-suited towards that direction. However, several of the devices discussed either exclusively or partly use non-standard processes and materials that are often not compatible with microelectronics processes. Additive manufacturing approaches and the use of soft materials present challenges in this direction. Thus, the proposition and design phase of such devices should take the above into consideration from an early stage and aim, where possible, for the use of materials and processes that take advantage of such mature processes and approaches.

Another issue that should be further considered is related to real-time sensing and readout accuracy. The readout device should provide a well-defined environment for the readout depending on the sensing approach and modality used. For example, alignment and distance limitations between the implanted and external primary device should be well-defined and controlled to ensure proper implant interrogation. Signal degradation in wireless communication through the tissue is well documented and extensively studied, but represents a challenge that needs to be taken into account [213,214,215,216]. While this is, to some extent, also common for batteryless active devices with active electronics, depending on how the information is encoded in the signal, sensing accuracy can be impacted in fully passive devices as they are fully analog implementations and there is no digital encoding in the sensing signal that is wirelessly obtained. Consequently, motion artefacts can impact real-time measurement accuracy. This is particularly true if the information is encoded in the amplitude of the signal. The lack of signal processing in the implanted device creates additional challenges for the readout device and for the overall system’s limit of detection and noise, common-mode rejection (CMR) and amplification requirements. The impact of the limitations of the readout device is often neglected in the literature. The presence of other devices and materials that can affect the coupling between the two devices and their resonance must also be considered. The use of magnets in both devices when possible can be exploited to ensure alignment, reduce motion artefacts and guarantee the distance between them [217,218,219,220]. 

Finally, it is important to highlight the challenges faced when moving from the lab-based prototype stage to the clinical application stage. Surpassing the so-called “valley of death” of medical devices, and moving from prototyping and proof-of-concept demonstration to clinical translation and into the hands of clinicians and patients to demonstrate real clinical impact, is a major challenge faced by all medical technologies [221,222,223,224,225]. Clinical trials and the necessary regulatory approvals present additional (necessary) obstacles that are not faced in other fields. As highlighted earlier, with the exception of the CardioMEMS device, to the authors’ knowledge, there has not been any other fully passive commercially available device. This highlights the difficulty in translating a prototype into a commercially viable and clinically useful product. A medical technology must not only satisfy regulatory and clinical requirements, it must also provide a clear advantage for healthcare systems and health insurers in terms of health economics in the long run [226]. Investors, similarly to regulatory bodies, need to see the technology’s efficacy and safety early in the development cycle. It is critical to have a clear roadmap to secure adequate funding at an early stage to be able to navigate the translational path and to move from basic research to clinical impact. Medical technology translation goes hand in hand with regulatory approvals. Clinical trials are complex and involve critical legal and ethical aspects. Apart from proving the efficacy of a solution, its safety and clinical impact, they are also valuable to obtain wider feedback and design input from the relevant stakeholders. Any solution must provide a clear value proposition addressing clinical needs and providing clinical impact more effectively than existing solutions, or addressing a clinical gap and unmet need. It is well-documented that a vast number of technologies do not survive past this valley of death, failing to provide reproducible data or real clinical impact.

Signal processing, statistical models and decision support systems (DSS) have the potential to enhance the readout quality and overall impact of fully passive implantable medical devices. Machine learning (ML) and artificial intelligence (AI) approaches are greatly impacting all fields of technology and our society as a whole, and thus such technologies can also impact the field of fully passive implantable devices, particularly with regards to the interpretation of recorded data and the identification of patterns in the data. Cloud computing for centralized ML of compressed sensing data, as well as event-based data recording and processing where relevant, are technologies that can also impact the use of passive sensors, as well as the use of multiparametric sensing implementations where sensor fusion can be exploited to enhance diagnostic capabilities. Digital twins have the potential of also aiding in the training of users and ML models, while also creating realistic test scenarios for the development of such sensing systems. 

Based on the strict definition given in the paper for fully passive sensors, these devices are zero-power devices, as they do not have any components requiring an energy source. It is the impact of the device properties that affect the properties of the external coupled system that is detected. Nevertheless, current advances in related technologies should also be mentioned for completeness. This includes energy-harvesting technologies that hold great promise for implanted medical devices. These include triboelectric nanogenerators (TENG), biofuel cells, piezoelectric energy harvesting and thermoelectric generators (TEG). Energy-harvesting approaches have the potential for creating self-powered implantable systems. An interesting recent example is the TENG-based implantable device proposed in [227], where micromechanical energy was harvested and converted into pulses for phrenic nerve stimulation. Implanted TENGs can also be energized using externally applied ultrasonic waves [228]. TENGs convert mechanical energy into electrical energy, exploiting triboelectric electrification and electrostatic induction using materials with distinct electronegativities. When two such materials come in contact and then the distance between them increases, one material gains while the other loses electrons. This leads to a potential difference being formed across the two materials. When the materials are laced back into contact, a current flows. Biodegradable implementations have been demonstrated with power densities in the region of 1087.6 mW/m^2^ [229,230]. TEGs are formed at the junction of two dissimilar materials. Implantable TEGs generate power by harnessing the human body’s temperature gradients, and devices generating power in the range of 110 μW have been reported [231]. The presence of a thermal gradient across their interface leads to a high-voltage output. Typically, 100–300 μV/K per junction can be obtained and several such junctions can be connected electrically in series and thermally in parallel to generate mWs of power from the human body. Nevertheless, the architecture of TEG devices is complex and requires materials and processes not easy to scale or easy to combine with standard fabrication processes and at-scale manufacturing [231,232]. The piezoelectric effect converts mechanical strain into electrical power. In an implantable application, this strain can be induced by the blood pulsating through a vein, or by a muscle or tendon [233]. Such piezoelectric devices employ ceramics and other non-standard materials. Blood glucose or other metabolites can be exploited in miniaturized two-electrode electrochemical biofuel cells to generate energy, either with the use of enzymes or not [42,234]. The lifetime of the enzyme is critical. Generated power in the region of 4300 μW cm^−2^ or less have been demonstrated, as summarized in [42], in human sweat, and more research is needed to demonstrate similar or greater power levels generated using interstitial and other biofluids. Depending on the source of energy and whether it is guaranteed, energy storage is typically needed. Biobatteries and supercapacitors have also been proposed in the context of fully biocompatible systems. The subject of biofluid-activated batteries and supercapacitors is rather new, and the limited available studies have demonstrated power densities in the order of 7460 μW cm^−2^ or less for biobatteries and in the order of 0.5 W cm^−2^ for supercapacitors using human sweat. In any case, power management and regulation that are based on active electronics are essential in energy-harvesting systems [235]. Coupled with lifetime issues, fabrication and integration challenges, increased complexity and issues arising from heterogeneous integration, apart from the limited power generation capabilities, such approaches, while promising, are still lagging behind other aforementioned approaches, while further motivating research and development of fully passive sensing systems. It is clear from the above that the field of passive sensors holds great promise for innovation and for technological solutions that can have a clinical impact. Several aspects of the necessary system-level solution are still in their infancy and a growing relevant ecosystem is needed that takes lessons from the microelectronics industry.

## Figures and Tables

**Table 1:** Indicative overview including several examples from the literature of fully passive, implantable, wireless sensors categorized by the detection quantity (sensor type). Information on the application, measurement scheme, communication and material type (transient or permanent) are also presented.

Sensor Type	Application	Sensor Scheme	Communication	Transiency	Ref.
pH	SSI	Speed of biodegradation process	Slot antenna at 1.5 GHz (max. size 5.1 cm)	Yes	[51]
pH	Soft-tissue trauma monitoring	Speed of biodegradation process	RFID tag, 4–11 GHz	Yes	[52]
pH	Detection of gastric leakage	LC circuit embedded in pH-responsive hydrogel	LC at 20 MHz	Yes	[53]
Pressure	Intra-abdominal pressure	Microfluidic channel with reservoir	Ultrasound imaging system (21–55 MHz transducer)	No	[54]
Pressure	Intracranial Dynamic Pressure Detection	Deformation of soft elastomer between two coils	LC between 200 and 500 MHz	Yes	[55]
Pressure	-	Capacitance, MEMS-type	LC at 30 MHz	Yes	[56]
Pressure	-	Capacitance, macroscopic MEMS-type	LC at 3 GHz	Yes	[57]
Pressure	Continuous arterial blood flow monitoring	Capacitance, MEMs-type and fringe-field	LC at 500 MHz	Yes	[58]
Pressure	Blood pressure monitoring on stents	Capacitance, MEMS-type	LC at 150 MHz	Yes	[59]
Pressure	Intracranial, intra-abdominal, and pulmonary hypertension, compartment syndromes	Capacitance, MEMS-type	LC at 250 MHz	Yes	[60]
Pressure	Blood pressure	Capacitance, MEMS-type	SAW at 0.87 GHz	No	[61]
Strain	Cardiomyocyte detection	Magnetic hysteresis loops	Vibrating sample magnetometer (VSM)	No	[62]
Strain	Orthopedic implants	Microfluidic channel with reservoir	Commercial medical ultrasonic device	No	[63]
Strain	Musculoskeletal soft tissue strains	Stretchable capacitor	LC at 15 MHz	No	[64]
Strain	Long-bone fracture healing	Stretchable capacitor	SRR at 530 MHz	No	[65]
Glucose	Continuous glucose monitoring	Capacitance, Volume change of glucose-sensitive hydrogel	RF ID (LC) 400–800 MHz	No	[66]
Glucose	Continuous glucose monitoring	Capacitance, dielectric constant of interstitial fluid	RF at 2.2 GHz	No	[67]
Tissue conductivity	Congestive heart failure	Discharging of capacitor	Alternating current bursts >1 MHz	No	[68]
Proteolytic activity	Healthcare applications	Capacitance, dielectric constant change by gelatin degradation	RF (LC) at ~15 MHz	Yes	[69]
Temperature	SSI	Ultrasound reflection	Ultrasound reflection at 5 MHz	No	[70]
Temperature	Regional Body Temperature	Capacitance, dielectric constant change due to temperature	LC at 70–90 MHz	Yes	[71]
Neural Recorder	Neuropotentials	Capacitance, varactor	RF at 2.32 GHz	No	[72]
